# Accelerometry and physical activity questionnaires - a systematic review

**DOI:** 10.1186/s12889-016-3172-0

**Published:** 2016-06-16

**Authors:** Stephanie Skender, Jennifer Ose, Jenny Chang-Claude, Michael Paskow, Boris Brühmann, Erin M. Siegel, Karen Steindorf, Cornelia M. Ulrich

**Affiliations:** Division of Preventive Oncology, National Center for Tumor Diseases (NCT), Im Neuenheimer Feld 460, 69120 Heidelberg, Germany; Division of Preventive Oncology, German Cancer Research Center (DKFZ), Im Neuenheimer Feld 460, 69120 Heidelberg, Germany; Division of Clinical Epidemiology, German Cancer Research Center (DKFZ), Im Neuenheimer Feld 581, 69120 Heidelberg, Germany; Department of Cancer Epidemiology, H. Lee Moffitt Cancer Center and Research Institute, 12902 Magnolia Drive, Tampa, FL 33612 USA; Unit of Environmental Epidemiology, German Cancer Research Center (DKFZ), Im Neuenheimer Feld 581, 69120 Heidelberg, Germany; Department of Population Health Sciences, University of Utah, Williams Building, 295 Chipeta Way, Salt Lake City, UT 84108 USA; Population Sciences, Huntsman Cancer Institute, University of Utah, Circle of Hope Dr. 2000, Room 4165, Salt Lake City, UT 84112 USA

**Keywords:** Accelerometry, Questionnaires, Physical activity measurement, Adults

## Abstract

**Background:**

The aim of this study is to review accelerometer wear methods and correlations between accelerometry and physical activity questionnaire data, depending on participant characteristics.

**Methods:**

We included 57 articles about physical activity measurement by accelerometry and questionnaires. Criteria were to have at least 100 participants of at least 18 years of age with manuscripts available in English. Accelerometer wear methods were compared. Spearman and Pearson correlation coefficients between questionnaires and accelerometers and differences between genders, age categories, and body mass index (BMI) categories were assessed.

**Results:**

In most investigations, requested wear time was seven days during waking hours and devices were mostly attached on hips with waist belts. A minimum of four valid days with wear time of at least ten hours per day was required in most studies. Correlations (r = Pearson, ρ = Spearman) of total questionnaire scores against accelerometer measures across individual studies ranged from *r* = 0.08 to *ρ* = 0.58 (*P* < 0.001) for men and from *r* = −0.02 to *r* = 0.49 (*P* < 0.01) for women. Correlations for total physical activity among participants with ages ≤65 ranged from *r* = 0.04 to *ρ* = 0.47 (*P* < 0.001) and from *r* = 0.16 (*P* = 0.02) to *r* = 0.53 (*P* < 0.01) among the elderly (≥65 years). Few studies investigated stratification by BMI, with varying cut points and inconsistent results.

**Conclusion:**

Accelerometers appear to provide slightly more consistent results in relation to self-reported physical activity among men. Nevertheless, due to overall limited consistency, different aspects measured by each method, and differences in the dimensions studied, it is advised that studies use both questionnaires and accelerometers to gain the most complete physical activity information.

**Electronic supplementary material:**

The online version of this article (doi:10.1186/s12889-016-3172-0) contains supplementary material, which is available to authorized users.

## Background

Physical activity (PA) is related to a number of health outcomes. According to the Global Recommendations on Physical Activity for Health by the World Health Organization (WHO), physical inactivity is the fourth leading risk factor for all deaths, and regular participation in PA reduces the risk of coronary heart disease and stroke, diabetes, hypertension, depression, breast and colon cancer [1].

PA is defined as any bodily movement that results in energy expenditure [2]. Aerobic, muscle-strengthening, bone-strengthening activity, and stretching are the four main types of PA [3]. It is a complex behavior and, thus, challenging to measure. Different methods to measure PA exist, including behavioral observations, questionnaires, PA diaries, direct/indirect calorimetry, and motion sensors, such as accelerometer, heart rate monitors (HRM), combined heart rate and accelerometry devices and pedometers. Due to the many different methods available to measure PA, there is a lack of comparability among studies. Furthermore, a number of challenges need to be considered for the various methods including expense, time, recall-bias and equipment needs. While the doubly labeled water (DLW) method is the most costly measurement, the most cost-effective measurement is the administration of PA questionnaires, which can assess all types of PA and can be used in large samples. They can also cover longer time frames which, however, may also lead to recall bias. PA questionnaires have been generally designed to minimize these potential biases as much as possible. For example the Global Physical Activity Questionnaire (GPAQ) asks about a “typical week” to reduce the need for longer recall [4].

Due to the complex and subjective information collected PA questionnaires may also over- or underestimate participants’ PA [5, 6]. In particular, older adults are more likely to engage in light- to moderate-intensity PA, which is the most difficult type of activity to be assessed by questionnaires [7]. Motion sensors, such as pedometers or accelerometers are increasingly implemented as an additional measure of PA in a free-living environment. Accelerometry has become a common tool in recent studies [8]. Accelerometers are small electronic devices that record acceleration associated with body movement and provide an objective estimate of duration and intensity of locomotion [9]. Today, a multitude of different accelerometers from a number of companies are on the market. They are generally able to assess PA in at least three axes (vertical, horizontal, and perpendicular). A typical output of accelerometer measurements is expressed in activity counts per unit of time, most frequently, counts per minute. In order to make the data comparable across types of accelerometers or types of PA measurement, activity counts can be translated into quantitative estimates of energy expenditure [10]. Each accelerometer model has its own algorithm to convert accelerometry counts into kilocalories (kcals) or metabolic equivalent of tasks (METs). This may lead to different output values depending on the model used so that one cannot directly compare data from different models. Accelerometers are designed to measure all PAs, however, they have also limitations. Depending on the attachment site, single accelerometers are not able to detect all movements, (e.g., upper/lower body or stationary movement) or capture the context in which the measured activities take place (e.g., leisure time or work). They are not suitable for long-term measurements, hence, repeated administration of accelerometers is of great importance in order to assess seasonable variation in PA. Water-based activities may also lead to misclassifications of an individual’s PA profile, because not all devices are waterproof, thus must be removed during such activities. Their administration is logistically more complex and costly.

In a publication on best practices of PA monitors by Matthews et al., the authors state that there is a variety of possible wear positions and that a wear-period of 7 days may be sufficient [11]. However, they suggest that further research is needed to inform the appropriate wear-time. Additionally, the daily required wear time is of strong interest, as it has been shown that modifications lead to significant differences in PA measures and adherence [12, 13]. Data collection is very dependent on compliance by the participant to wear the device. Prior research indicates that healthy participants who are younger, unemployed or current smokers are more likely to be noncompliant [14]. This variance in compliance is less likely to occur in cancer patients, as these are often motivated to modify their lifestyles [15]. This is underlined by the results of a prior study in colorectal cancer patients that reported no significant differences in compliance of wear-time by age, gender, BMI or tumor stage [16].

Many recent investigations combined the use of questionnaires and motion sensors in order to collect complementary and comprehensive data. Several systematic reviews have been performed comparing objective versus self-reported PA [17, 18]. However, these reviews focused on different domains compared to this presented review. Prior research articles and questionnaires predominantly focused on a specific type of PA (e.g., leisure time, work). The GPAQ, however, is a more recently used instrument to assess several types of PA and thus, may be able to provide a more complete impression of an individual’s level of PA. It may be of future interest to review correlations between multimodal PA questionnaires such as the GPAQ and accelerometer data, when this method has been applied more often.

The objective of this study is to review accelerometer settings and wear methods to determine whether a practical standard for settings/wear methods exists. Furthermore, the aim is to determine correlations between accelerometry and PA questionnaire data, overall, and by gender, age and BMI. We believe that our approach using a large set of studies and stratifying on specific subgroups helps to fill gaps in our understanding of PA assessment types in multiple populations.

## Methods

A total of 57 full articles published on simultaneous PA measurement in adults with accelerometry and questionnaires in free-living conditions were reviewed. A literature search was conducted in PubMed in July 2014. Search terms included “accelerometry”, “accelerometer”, “accelerometers”, “motion sensor” or “motion sensors”, and “questionnaire” or “questionnaires” and had to be identified in the title or abstract. For all publications, the following inclusion criteria were applied: (1) All participants within each study had to be adults (18+ years) to reduce age-related differences in PA patterns and (2) relevant investigations had to include a sample size of at least 100 participants to increase stability of the observed associations and to allow investigation of differences by age, sex and body mass index (BMI). The following exclusion criteria were applied to improve study comparability: (1) studies with wheelchair-using or non-ambulatory participants (2) articles not available in English, and (3) investigations lacking correlation data between accelerometry and questionnaires. Two authors (SS, MP) independently screened and extracted data from the studies according to the above mentioned criteria, regardless of publication date. Disagreements were discussed between the two authors and then resolved.

For this review, all investigations providing correlational comparison values between the different types of PA measurements for the purpose of validity assessment are presented and distinguished by sex, age, and BMI categories where possible.

Both, Spearman and Pearson correlation coefficients were included, depending on which type was provided by the study. The main difference between these two measures is that the Spearman correlation coefficient applies to non-parametric data, whereas the Pearson correlation coefficient requires normally distributed data. In order to make it clear to the reader which coefficient has been used we present (ρ) for Spearman correlations and (r) for Pearson correlations. Reported metrics in this review were derived from the individual studies. Correlations of accelerometer-derived total PA and total measures from questionnaires assessing sedentary behavior were excluded as they are expected to be inversely correlated. Accelerometer wear methods were also extracted. In Additional files 1, 2 and 3, the number of participants is presented along with the brand and/or model of accelerometer used in the investigations, the settings of the accelerometer measurements, the questionnaires used, and the correlations between PA measured by questionnaire accelerometer. Results organized by sex, age and BMI categories are presented sequentially.

## Results

**Fig. 1:**
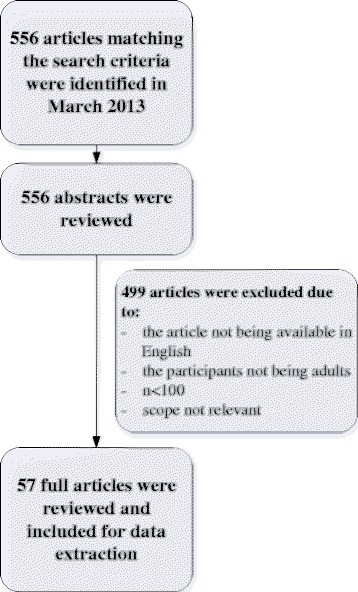
PRISMA flow diagram [90]: Search strategy and filter by exclusion criteria

The defined search strategy in PubMed resulted in 556 records (Fig. 1). All abstracts were reviewed and after application of the inclusion and exclusion criteria, 57 investigations remained relevant for this review (Table 1, sorted by publication date). Main exclusion criteria for this manuscript were lack of information on correlations, fewer than 100 participants, age younger than 18 years, and non-English publication (Additional file 4).

**Table 1:** Publications included in this review (*n* = 57)

Author	Journal	Year
Sabia	Am J Epidemiol. 2014 Mar 15;179(6):781–90.	2014
Dahl-Petersen	Med Sci Sports Exerc. 2013 Apr;45(4):728–36	2013
Segura-Jiménez	Clin Exp Rheumatol. 2013 Nov-Dec;31(6 Suppl 79):S94–101. Epub 2013 Dec 2	2013
Pettee Gabriel	Menopause. Feb 2013; 20(2): 152–161.	2013
Warner	Am J Health Behav. Mar 2012; 36(2): 168–178.	2013
Hekler	J Phys Act Health. 2012 Feb;9(2):225–36.	2012
Kwak	J Phys Act Health. 2012 Nov;9(8):1130–7. Epub 2011 Dec 27	2012
Celis-Morales	PLoS ONE 7 (5): e36345. 2012 May 9	2012
Scheers	Int. Journal of Behavioral Nutrition and Physical Activity. 9:71. 2012	2012
Dunton	Front Psychol. 2012; 3: 260.	2012
The InterAct Consortium	Eur J Epidemiol (2012) 27:15–25	2012
Sullivan	Int J Behav Nutr Phys Act. 2012; 9: 13.	2012
Grimm	J Aging Phys Act. 2012 Jan;20(1):64–79	2012
Mâsse	J Phys Act Health. 2012 Feb;9(2):237–48	2012
De Hollander	J Clin Epidemiol. 2012;65(1):73–81	2012
Nang	BMC Med Res Methodol. 2011 Oct 13;11:141	2011
Semanik	Arthritis Care Res (Hoboken) 2011 Dec; 63(12):1766–72. doi: 10.1002/acr.20644	2011
Lee	J Community Health. 2011 Dec;36(6):1011–23. doi: 10.1007/s10900-011-9403-5.	2011
Tomioka	J Epidemiol. 2011 Nov 5; 21(6):459–65. Epub 2011 Sep 24	2011
Lee	Int J Behav Nutr Phys Act. 2011 Aug 1; 8:81.	2011
Peters	Med Sci Sports Exerc. 2010 Dec; 42(12):2222–30.	2011
Clark	Med Sci Sports Exerc. 2011 Oct; 43(10):1907–12.	2011
Lee	J Community Health; 26 April 2011	2011
Nicaise	Journal of Physical Activity and Health; 8, 881–890. 2011	2011
Emaus	Scand J Public Health. 2010 Nov; 38(5 Suppl):105–18.	2010
Hagstromer	J Phys Act Health. 2010 Jul;7(4):541–50	2010
Weikert	J Neurol Sci. 2010 Mar 15; 290(1–2):6–11. Epub 2010 Jan 8.	2010
Hallal	Journal of Physical Activity & Health, 2010, 7, 402–409	2010
van der Ploeg	Research Q Exercise Sport, 2010, Vol. 81, No. 1,97–101	2010
Rosenberg	Journal of Physical Activity and Health, 2010, 7, 697–705	2010
Wollmerstedt	J Arthroplasty. 2010 Apr;25(3):475–480	2010
Evenson	Intl Journal of Behavioral Nutrition and Physical Activity. 2010. 7:21	2010
Li	Eur J Clin Nutr. 2009 Dec; 63(12):1448–51. Epub 2009 Jul 29.	2009
Sloane	Med Sci Sports Exerc. Jun 2009; 41(6): 1334–1340.	2009
Harris	Med Sci Sports Exerc. 2009 Jul;41(7):1392–402	2009
Jacobi	Eur J Epidemiol. 2009; 24(4):171–9. Epub 2009 Mar 13.	2009
Cust	Epidemiology. 2009 May; 20(3):433–41.	2009
Trinh	Journal of Physical Activity and Health, 2009, 6(Suppl 1), S46–S53	2009
Bull	Journal of Physical Activity and Health, 2009, 6, 790–804	2009
Hagiwara	Geriatr Gerontol Int. 2008 Sep; 8(3):143–51.	2008
Wolin	Br J Sports Med. 2010 Aug; 44(10):741–6. Epub 2008 Nov 3.	2008
Motl	Ann Behav Med. 2008 Aug; 36(1):93–9. Epub 2008 Aug 22.	2008
Rosenberg	J Phys Act Health. 2008; 5 Suppl 1:S30–44.	2008
Cust	Intl Journal of Behavioral Nutrition and Physical Activity. 2008. 5:33	2008
Orsini	Eur J Epidemiol (2008). 23: 661–667	2008
Yasunaga	Journal of Aging and Physical Activity, 2007, 15, 398–411	2007
Carter-Nolan	Ethn Dis. 2006 Autumn; 16(4):943–7.	2006
Ekelund	Public Health Nutr. 2006 Apr; 9(2):258–65.	2006
Friedenreich	Am J Epidemiol. 2006 May 15; 163(10):959–70. Epub 2006 Mar 8	2006
Johnson-Kozlow	Intl Journal of Behavioral Nutrition and Physical Activity. 2006. 3:7	2006
Kolbe-Alexander	J Aging Phys Act. 2006 Jan;14(1):98–114	2006
Gardner	Vasc Endo-vascular Surg. 2006 Oct-Nov; 40(5):383–91.	2006
Smith	Am J Prev Med. 2005 Nov;29(4):256–64	2005
Timperio	Med Sci Sports Exerc. 2004 Jul;36(7):1181–6.	2004
Timperio	J Sci Med Sport. 2003 Dec; 6(4):477–91.	2003
Craig	Med Sci Sports Exerc. 2003 Aug; 35(8):1381–95.	2003
Philippaerts	Int. J Sports Med 2001; 22: 34–39	2001

Comparisons between questionnaires and accelerometers were reported as Spearman (ρ) or Pearson (r) correlation coefficients as reported in the original manuscripts. In one study, by DeHollander et al. [19], no correlations were reported, instead, percentage of exact agreement and maximum agreement were calculated.

### Accelerometer wear methods: wear-time and application

In 37 of the 57 reviewed investigations, participants were asked to wear the accelerometers for 7 consecutive days during waking hours [20–56]. Eight investigations asked participants to wear the accelerometers for 4 days [19, 43, 57–62]. In only 4 studies participants were asked to wear the accelerometers for more than 7 days (9 days to four weeks) [63–66]. For the rest of the studies, wear-time ranged between 2 and 6 days [67–72]. In nearly all reviewed studies, participants wore the devices only during waking hours and removed them during water-based activities. Many of the investigations (*n* = 14) required a minimum of 4 valid measurement days per week [19, 29, 30, 33, 35, 40, 41, 45, 56–58, 65, 70, 72]. Nine studies required a wear-time of at least 5 days, and 7 investigations reported various minimum numbers of days ranging between 1 and 7 days [21, 23–28, 34, 36, 38, 39, 44, 50, 62, 66, 69]. Definitions of ‘valid days’ were available in 28 of the investigations; 23 of these studies required a minimum wear-time of ten hours per day [21, 23, 24, 26, 27, 30, 31, 33, 34, 36, 38–41, 43–45, 47, 50, 56, 57, 69, 70, 72], one deemed eight hours per day to be sufficient for the day to be valid [29], one 22 h per day [68], one 16 h [65] and 2 others even 24 h [19, 50]. Spearman correlations tended to be stronger with increased wear-time (over 7 days). When wear-time accounted for at least 14 days, correlations ranged from *ρ* = 0.41 to *ρ* = 0.53 [63, 64]. One exception were the results from the investigation conducted by Gardner et al. [67] where wear-time was only 2 consecutive days and Pearson’s correlation between the questionnaire and accelerometry was reported as *r* = 0.71.

In almost all investigations which reported the placement of the accelerometer participants wore the devices attached to an elastic belt. One study preferred attachment with a clip, but provided waist belts in case the clip attachment was not possible [70]. Of the studies that specified the placement of accelerometers, 17 reported attachment on the hip [20, 28, 30, 31, 33–35, 39, 46–50, 57, 64, 69, 70, 72] and 6 studies reported attachment around the waist [29, 32, 39, 43, 60, 73]. Chest [58], iliac crest [35], wrist [65] and triceps [68] were also reported attachment sites.

The 57 studies reviewed investigated correlations of questionnaire-derived PA measures with accelerometry-derived PA measures. Many investigations used the International Physical Activity Questionnaire (IPAQ), however, there was still a great diversity of questionnaires used in the different studies. Most of the reviewed studies used an Actigraph (formerly known as MTI/CSA) monitor. Overall correlations for total PA ranged from *r* = 0.14 (*P* < 0.001) [52] to *r* = 0.58 (*P* < 0.001) [69]. Of the reviewed studies only one third reported correlations ≥0.40.

**Fig. 2:**
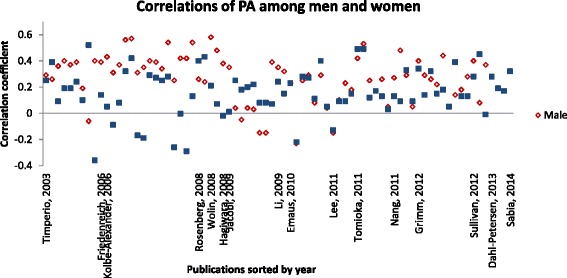
Correlations among men and women within the individual studies

Gender comparisons were available in 25 of the included studies (Additional file 1) [20, 22, 24, 27, 29, 31, 39, 42, 43, 45, 47–49, 52, 53, 56, 57, 61, 62, 64, 65, 69–71, 73] and seven were limited specifically to women [22, 45, 49, 50, 53, 66, 74]. Correlations of total questionnaire scores with total accelerometer measures ranged from *ρ* = 0.08 (n.s.) [31] to *r* = 0.58 (*P* < 0.001) [70] for men and from *r* = −0.02 (n.s.) [71] to *ρ* = 0.49 (*P* < 0.01) [64] for women. Of the 21 studies that reported correlations by gender, most investigated associations for moderate and vigorous intensity PA. For moderate PA data from questionnaires and accelerometers, correlations ranged from *r* = 0.03 (n.s.) [29] to *r* = 0.40 (*P* < 0.01) [20, 47] for men and from *r* = −0.02 (n.s.) [53] to *r* = 0.29 (*P* = 0.02) [47] for women. Correlations for vigorous PA ranged from r = −0.15 (n.s.) [29] to *r* = 0.43 (*P* = 0.05) [47] for men and from *r* = −0.36 (*P* < 0.05) [20] to *r* = 0.52 (*P* <  0.001) [20] for women. Associations for moderate-to-vigorous intensity PA among men and/or women were examined by 6 investigations [20, 31, 42, 48, 49, 57] and ranged from *ρ* = 0.04 (n.s.) [31] to *r* = 0.44 (*P* < 0.01) [48] for men and from *ρ* = 0.01 (n.s.) [57] to *r* = 0.39 (*P* < 0.01) [20] for women. Emaus et al. [31] and Sullivan et al. [57] reported correlations for light PA between *ρ* = −0.23 (*P* < 0.05) and *ρ* = 0.29 (*P* < 0.01) among men and *ρ* = −0.22 (*P* < 0.05) and *ρ* = 0.45 (*P* < 0.05) among women. Within the individual studies, associations between measured and reported PA tended to be stronger among men than among women (Fig. 2).

Six studies investigated PA correlations distinguished by BMI categories (Additional file 2) [20, 24, 41, 43, 52, 72]. There were no consistently defined cut points for BMI among the 6 investigations. Cust et al. [41] used median BMI as cut point <27.2 kg/m^2^ and ≥27.2 kg/m^2^. Association of PA assessed by questionnaire and measured by accelerometer were stronger for the lower BMI group, although differences were not statistically significant. Lee et al. [43] and Friedenreich et al. [24] set the cut points to <25 kg/m^2^and ≥25 kg/m^2^ which is defined as the standard by the WHO for distinguishing overweight/obese from normal-/underweight individuals [75]. Correlations ranged from *ρ* = 0.09 (*P* < 0.01, moderate intensity PA) [43] to *r* = 0.38 (*P* < 0.05, total PA) [24] for the group with lower BMI and from *ρ* = 0.10 (n.s., moderate intensity PA) to *ρ* = 0.22 (*P* < 0.001, vigorous intensity PA) [43] for the group with higher BMI. Warner et al. [72] and Kwak et al. [52] set their cut points to <25 kg/m^2^ (normal), ≥ 25- < 30 kg/m^2^ (overweight) and ≥30 kg/m^2^ (obese). Whereas Warner et al. observed the strongest correlations between questionnaire-derived total PA and accelerometry among overweight participants (*r* = 0.55, *P* = 0.003), Kwak et al. noted the strongest correlations regarding work-related PA among normal weight participants (*r* = 0.46, *P* < 0.01). The results from Lee et al.[43] showed stronger, yet statistically non-significant, correlations for participants with a BMI ≥25 kg/m^2^ whereas Friedenreich et al. [24] reported stronger associations for the group with lower BMI (BMI <25 kg/m^2^). Timperio et al. [20] set the cut points for BMI to ≤25 kg/m^2^ and >25 kg/m^2^ and also stratified results by gender. Among participants with a BMI ≤25 kg/m^2^ correlations ranged from *r* = −0.06 (n.s., men) for intensities of ≥6 METs to *r* = 0.52 (*P* ≤ 0.001, women) for intensities of ≥6 METs. Correlations among participants with a BMI >25 kg/m^2^ ranged from *r* = −0.36 (*P* < 0.05, women) to *r* = 0.40 (*P* < 0.05, men). Overall, no consistent results according to BMI could be identified in these four studies.

Regarding age of participants, only the results of 17 studies could be compared due to differences in setting the cut points for age. For this review, a cut point of 65 years of age was chosen in order to capture the greatest possible amount of studies. The age of 65 years is also a generally accepted cut point in developed countries to distinguish between the elderly and the younger, commonly working population.

Results from eleven investigations could be summarized for participants ≤65 years of age (Additional file 3) [21, 24, 30, 35, 36, 41, 43, 44, 59, 65, 68, 69]. Correlations ranged from *ρ* = 0.04 (n.s.) [43] to *r* = 0.47 (*P* < 0.001) [59] for total PA. For moderate intensity, correlations between PA assessed by questionnaire and accelerometry ranged from *r* = 0.01 (n.s.) to *ρ* = 0.35 (95 % CI = 0.08–0.55) [30], and for vigorous intensity, correlations ranged from *r* = −0.03 (n.s.) [36] to *r* = 0.83 (*P* < 0.05) [69]. Two studies also reported associations for moderate-to-vigorous PA [30, 36]. The weakest association was *r* = 0.09 (n.s.) and the strongest was *r* = 0.30 (95 % CI = 0.09–0.49). Five studies reported results for the elderly of ≥65 years of age [28, 63–65, 71]. Correlations ranged from *r* = 0.16 (*P* = 0.02) to *r* = 0.53 (*P* < 0.01) for total PA. Tomioka et al. [64] also reported correlations for vigorous and moderate PA. For vigorous intensity, the weakest association was seen among women from 65 to 74 years of age [*ρ* = 0.12 (n.s.)] and the strongest was seen among men from 65 to 74 years of age (*ρ* = 0.25, *P* < 0.05). For moderate PA the weakest correlation was *ρ* = 0.03 (n.s.) among women from 75 to 89 years of age and the strongest was *ρ* = 0.26 (*P* < 0.05) among men from 65 to 74 years of age. Yasunaga et al. [63] assessed associations for moderate-to-vigorous and light PA among the elderly (≥65 years of age), with correlation coefficients of *r* = 0.53 (n.s.) and *r* = 0.28 (n.s.), respectively. Although these results showed no consistency among age groups, correlations within the individual studies tended to be slightly higher for younger age groups.

## Discussion

This review identified 57 publications that compared PA questionnaires with accelerometry data. Although there have been a few systematic reviews discussing self-reported versus objective PA [17, 18], the present work is novel both in its subgroup assessment as well as in its framing of accelerometer wear methods. Today there are no set standards for use of accelerometers with respect to wear-time, minimal wearing time to be considered valid, or position of application, even though there seem to be trends for each of these aforementioned elements. Large observational studies, such as the National Health and Nutrition Examination Survey (NHANES) study have changed their protocols from attachment on hips to wrists [76]. Some studies suggest that hip-worn accelerometers assess PA more precisely compared to wrist-worn devices [77] whereas other investigations reported reasonable precise estimations of PA when using wrist-worn devices [78, 79]. However, to some extent wear methods are dependent on the study aim, the design of the accelerometers or the activity that is aimed to be captured, as well as acceptability within the study population. Of the 57 studies reviewed, accelerometer wear-time of 7 consecutive days during waking hours was the most consistently reported duration of measurement (*N* = 37, 65 %). Further requirements included having at least four out of 7 valid days (14 out of 37 studies), which was defined in most investigations, as being worn for at least 10 h. Many studies included at least one weekend day of the required wear-period. Since correlations seemed to be stronger with increased wear-time one could consider longer accelerometer wear-time for future studies. Additionally, in previous studies it could be shown that altering wear time led to significant differences in adherence as well as PA measures [12, 13]. It is important to note that in this review wear-time information was investigated only in the 57 included studies, and not in all available studies using accelerometry. Nevertheless, the identified inconsistencies in wear-time requirement within the 57 investigations demonstrate the need for general guidelines for the use of accelerometers in free-living conditions in order to increase comparability of these and future studies.

It has been shown that healthy, younger, unemployed and smoking participants are less likely to be compliant regarding wearing-time of accelerometers while participants that are suffering from a serious disease may be more interested in participating in research and thus may be more compliant.

Investigations reviewed in this manuscript compared PA scores of questionnaires with PA measures from accelerometers. In the 57 investigations, correlations between questionnaires and accelerometry were weak to moderate. This finding is in agreement with previous reviews [80, 81]. Potential explanations for this result might be associated with the advantages and disadvantages of both methods. Questionnaires can assess all types of PA, including stationary activities such as weight lifting. They can also cover long time frames. However, due to the complex and subjective nature of the gathered information, they may be subject to limitations in recollection or to recall bias, such as estimating or recalling the incorrect intensity [5, 6, 82]. Alternatively, accelerometers assess PA continuously and objectively. Unlike questionnaires, they are not suitable for long-term measurements and thus seasonable activities can be captured only through repeated administration. This is expected to reduce correlations. There are further aspects that limit PA measurement by accelerometry, such as the devices not being able to cover stationary activities, strength training, or cycling. Water-based activities can also lead to misclassifications in individual PA measurement in cases where the sensors are not waterproof or not worn during that activity. In addition, the wearing of an accelerometer itself may promote PA [16].

Data retrieved from accelerometers are commonly expressed as “counts”. This non-dimensional unit cannot be meaningfully interpreted, and therefore there exists the need to convert counts to an informative measure of PA, such as METs or kilocalories (kcals). With the help of regression equations, accelerometer counts are translated into measures of energy expenditure and measured PA can be classified into different intensities. There are many different regression equations reported, and depending on which accelerometer was used to determine the amount and intensity of PA, correlations with questionnaires vary, as different data processing algorithms result in different values of PA outcome measures [10]. Bassett et al. [83] reported that accelerometers may over-predict energy expenditure during walking while they may under-predict energy expenditure of many other activities. In the 57 studies, accelerometry data was reported as MET scores, time spent in physical activities, accelerometer counts per minute, or step counts. Questionnaire data was also reported in various measures (e.g., minutes per day, hours per week). This variation limits the ability to compare results across studies. Data processing guidelines for accelerometry would allow comparability among studies.

Among 25 studies, vigorous activity was more strongly correlated with self-report in men than in women ((e.g., *r* = 0.43, (*P* < 0.05) men vs. *r* = 0.05, (n.s.) women [47] or *ρ* = 0.23, *P* < 0.001 men; *ρ* = 0.09, *P* < 0.05 women [43])). This could be explained by the fact that men have higher levels of vigorous PA [24, 84], which is more easily assessed by questionnaires. Women tend to engage more in light PA, which is the most challenging type of activity to recall because it is most dominant in daily life as, for example, in household activities [24]. Correlations for light physical activities were investigated by Emaus et al. [31]. They showed negative correlations for self-reported and objectively measured leisure activities with light PA among both men and women (*ρ* = −0.23, *P* < 0.05 and *ρ* = −0.22, *P* < 0.05), whereas weak to moderate correlations were reported for work activities (*ρ* = 0.29, *P* < 0.01 men and *ρ* = 0.40, *P* < 0.001 women).

Only 6 studies investigated PA correlations by BMI categories and of those, there were small differences in defining the BMI categories, thus making comparison across studies challenging. The four investigations presented their results in different manners concerning BMI categories as well as different PA intensity categories. This inconsistency, once again, shows the importance of general guidelines to enable a reasonable comparison across studies like this.

Although there were no conclusive findings suggesting stronger associations by age group (<65 years vs. ≥65 years) correlations tended to be slightly higher among participants in the younger groups. Notably, most PA questionnaires are designed for younger populations; the focus of these studies is more on sports and recreational activities and therefore, do not meet the criteria for the elderly.[85] Kowalski et al. investigated the agreement between objective and self-reported PA in older adults and found generally weak to moderate correlations (*r* = −0.02–0.79) [17]. Older people are more likely to engage in activities that are most inaccurately assessed by questionnaires [7]. This might be an explanation for the slightly weaker correlations among the elderly reported here.

While studies assessing the correlations between questionnaire and accelerometer data are the primary focus of this review, an alternative method to assess agreement between two quantitative measurements are Bland-Altman plots [86]. Prior research has shown that bias in questionnaires can be revealed by Bland–Altman plots, while it may remain undetected by the use of correlation coefficients [87]. Therefore, studies using this graphical method may provide additional valuable insights [88]. Of the 57 studies presented in this review 18 utilized Bland-Altman plots to evaluate agreement between the mean differences of questionnaire and accelerometer data [21, 23, 28, 35, 36, 40, 42, 47, 51, 52, 56, 57, 60, 62, 68–70, 73]. We present the results of two studies exemplary. Interested readers are advised to consult the references provided above. Dahl-Petersen and colleagues [62] reported the results of Bland-Altman agreement methods and correlation coefficients. They observed moderate validity for questionnaire-based overall PA from the IPAQ compared to accelerometer data (*r* = 0.20–0.35, *P* < 0.01). Bland-Altman agreement analyses showed relatively small median differences for all measures of PA; however, moderate-intensity PA was substantially greater when reported by IPAQ when including walking [62]. Similarly, a study in an Asian population [69] showed a higher estimate of self-reported PA using the IPAQ compared to accelerometer data. These examples illustrate that, beyond correlation coefficients the Bland-Altman method provides additional information on the agreement between questionnaires and accelerometers.

The strength of this review is the inclusion of more than 50 studies with at least 100 participants which results in increased stability of observed associations. However, the varying measurement conditions and methods complicate comparison of findings from different studies. The reported accelerometry metrics in this review are derived from the individual studies and thus can differ. Questionnaires that assess PA are variable, with differences in number of items, time frame, focus, or background and characteristics of study population, which further complicates comparisons among different studies. A further limitation is that information on the exclusion of PA bouts of less than 10 min, which can have a significant effect on the correlations, was not always available. In this review, only the available correlational information from each investigation was used to compare results from the 57 studies in order to facilitate comparison among all reviewed studies. However, there are also other well-established methods to demonstrate associations (e.g., regression). Furthermore, due to the minimum number of required participants for inclusion into this review, most studies included healthy participants from the general population.

Only eight studies included participants with breast or prostate cancer, arterial diseases, multiple sclerosis, fibromyalgia, total hip arthroplasty, or rheumatoid/osteoarthritis [26, 32, 33, 67, 73, 89]. PA plays a significant role in the prevention or progression of different diseases [1]. This fact illustrates the importance of continued research of PA not only in healthy populations, but particularly in diseased cohorts in order to establish guidelines for patients or their physicians; Patients diagnosed with a disease such as cancer are often motivated to modify their lifestyles [15].

This review highlights the need for further research on the assessment of PA in studies. Due to the inconsistent correlations, the different aspects measured by questionnaires and accelerometers and some differences in the dimensions studied, future investigations should ideally use both questionnaires and accelerometers to gain the most accurate possible and complementary information. Needed are also guidelines for accelerometer settings, data processing and wear methods and the summaries presented in this review may help foster these. As reported in this review there were only a few studies investigating PA in diseased populations. Due to the importance of PA in the prevention of many diseases, such as cancer, more investigations relating to PA assessment in diseased populations are needed.

## Conclusion

There were no clear patterns in correlations between PA questionnaires and accelerometry by gender, age, BMI, or wear time. However, correlations seemed to be slightly stronger among men compared to women and younger vs. older populations. Due to differences in the dimensions studied by each method, it is advised that studies use both questionnaires and accelerometers to gain the most complete information. Furthermore, due to the low number of studies in patient groups, continued research to identify the best combination of wear methods is needed.

## Abbreviations

BMI, body mass index; DLW, doubly labeled water; GPAQ, Global Physical Activity Questionnaire; HRM, heart rate monitors; IPAQ, International Physical Activity Questionnaire; kcal, kilocalories; MET, metabolic equivalent of tasks; NHANES, National Health and Nutrition Examination Survey; PA, physical activity; WHO, World Health Organization

## Additional files

Additional file 1: Summary of correlations of physical activity data assessed by questionnaires and accelerometers with respect to gender. (PDF 179 kb) Additional file 2: Summary of correlations of physical activity data assessed by questionnaires and accelerometers with respect to BMI. (PDF 183 kb) Additional file 3: Summary of correlations of physical activity data assessed by questionnaires and accelerometers with respect to age. (PDF 263 kb) Additional file 4: PRISMA checklist [90]. (DOC 62 kb)
