# Supplementary material for: Accelerometry and physical activity questionnaires - a systematic review
Source: BMC Public Health. 2016 Jun 16;16:515. doi: 10.1186/s12889-016-3172-0 (PMC4910242; doi:10.1186/s12889-016-3172-0)
Supplement: Additional file 1: — Summary of correlations of physical activity data assessed by questionnaires and accelerometers with respect to gender. (PDF 179 kb) [file 12889_2016_3172_MOESM1_ESM.pdf]

Additional File 1. Summary of correlations of physical activity data assessed by questionnaires and accelerometers with respect to Gender

Study	***Journal***	***No. of Participants***	***Accelerometer used***	***Accelerometer wear method***	***Questionnaire used in investigation***	***Results***
**Timperio, 2003**	J Sci Med Sport. 2003 Dec; 6(4):477-91.	n= 144<br>n= 122 (for validation data)<br>18+ years	MTI/CSA accelerometer (model 7164)	7 consecutive days, during waking hours, attached with adjustable belt, on right hip, 1 minute epochs	One week recall PAQ, assesses frequency and duration of walking, other moderate intensity activity and vigorous intensity activity, questionnaire administered twice -> 3 days apart, again after accelerometer measurement	Spearman correlation<br>**Correlation PAQ (min/day) vs. Accelerometer (min/day)**<br>PAQ (duration of ≥3.0 METs) vs. Acc:<br>men / women<br>overall ρ= 0.29 $p < 0.05$ / ρ= 0.25 $p < 0.05$<br>BMI ≤25 ρ= 0.26 / ρ= 0.39 $p < 0.01$<br>BMI >25 ρ= 0.36 $p < 0.05$ / ρ= 0.09<br>PAQ (duration of 3.0-5.9 METs) vs. Acc:<br>men / women<br>overall ρ= 0.40 $p < 0.01$ / ρ= 0.19<br>BMI ≤25 ρ= 0.37 $p < 0.05$ / ρ= 0.19<br>BMI >25 ρ= 0.39 $p < 0.05$ / ρ= 0.24<br>PAQ (duration of 6.0+ METs) vs. Acc:<br>men / women<br>overall ρ= 0.19 / ρ= 0.10<br>BMI ≤25 ρ= -0.06 / ρ= 0.52 $p \leq 0.001$<br>BMI >25 ρ= 0.40 $p < 0.05$ / ρ= -0.36 $p < 0.05$
**Carter-Nolan, 2006**	Ethn Dis. 2006 Autumn; 16(4):943-7.	n = 101 African American women, residence in Washington DC, metropol. Area, 21-69 years ambulatory	Computer Science and Applications Model 7164 (version AM7164-2.2)	7 consecutive days, during waking hours, 1 min-epochs	Questions from BWHS (Black Women's Health Study), hours/week of participation in walking for exercise, hours/week of moderate activity, hours/week of strenuous activity	Spearman Correlation<br>**Acc counts - BWHS Qu**<br>ρ= 0.28[1] (BWHS total activities)<br>ρ= 0.26[1] (BWHS walking)<br>ρ= -0.04 (BWHS moderate activity)<br>ρ= 0.4[1] (BWHS vigorous activity)<br>[1] is significant at the 0.01 level

Additional File 1. Summary of correlations of physical activity data assessed by questionnaires and accelerometers with respect to Gender (continued)

Study	***Journal***	***No. of Participants***	***Accelerometer used***	***Accelerometer wear method***	***Questionnaire used in investigation***	***Results***
**Friedenreich, 2006**	Am J Epidemiol. 2006 May 15; 163(10):959-70. Epub 2006 Mar 8	n= 154, 35-65 years, residence in Calgary Health Region of Alberta, Canada	MTI ActiGraph (Manufacturing Technology Inc., Fort Walton Beach, FL, USA)	7 days during waking hours, 4 one week periods within 1 year, 12 weeks apart to cover all seasons during waking hours, min 3 days, min 10 hours/day	PYTPYQ (past year total physical activity qu), occupational, household, recreational activity, at baseline, 9 weeks after baseline, after 12 months	Spearman rank correlation<br>**PYTPAQ - Acc** (total PA)<br>ρ= 0.26 (total population) p <0.05<br>ρ= 0.39 (male) p <0.001<br>ρ= 0.14 (female)<br>ρ= 0.43 (age < 50) p <0.001<br>ρ= 0.05 (age ≥ 50)<br>ρ= 0.38 (BMI<25) p <0.05<br>ρ= 0.19 (BMI ≥ 25)<br>ρ= 0.26 (moderate PA) *significant<br>ρ= 0.34 (vigorous PA) *significant<br>ρ= -0.08 (light PA)
**Kolbe-Alexander, 2006**	J Aging Phys Act. 2006 Jan;14(1):98-114	n= 122, >60 years, 52 men, 70 women, from mixed-racial-ancestry population group	MTI (formerly Computer Science and Applications [CSA], Inc.] Model 7162, Shalimar, FL	7 consecutive days, 60s time epochs, during waking hours, on belt around waist, on right hip, min 10h/d	IPAQ (short version), intensity and duration of activities, transport, leisure and household activities, during usual week<br>YPAS (Yale Physical Activity Survey), part 1, frequency and duration in activities related to housework, yard work/gardening, caregiving, recreation and exercise, during week before interview	Spearman's rank order correlations<br>**IPAQ (MET-min) - accelerometer** (see table below)

		men		women	
IPAQ	Acc	ρ	P	ρ	P
vigorous	high counts time	0.43	0.05	0.05	
moderate	moderate	0.31	0.004	-0.09	
moderate	total counts time	0.37	0.017	0.08	
walking	moderate	0.56	0.0001	0.32	0.011
walking	total counts time	0.57	0.00007	0.42	0.0006
sitting	moderate continuous	-0.45	0.003	-0.22	
sitting	time	-0.39	0.01	-0.32	0.011
Weekend sitting	total counts	-0.40	0.001	-0.35	0.005

Additional File 1. Summary of correlations of physical activity data assessed by questionnaires and accelerometers with respect to Gender (continued)

Study	*Journal*	*No. of Participants*	*Accelerometer used*	*Accelerometer wear method*	*Questionnaire used in investigation*	*Results*
**Kolbe-Alexander, 2006 (continued)**	J Aging Phys Act. 2006 Jan;14(1):98-114	n= 122, >60 years, 52 men, 70 women, from mixed-racial-ancestry population group	MTI (formerly Computer Science and Applications [CSA], Inc.] Model 7162, Shalimar, FL	7 consecutive days, 60s time epochs, during waking hours, on belt around waist, on right hip, min 10h/d	IPAQ (short version), intensity and duration of activities, transport, leisure and household activities, during usual week<br>YPAS (Yale Physical Activity Survey), part 1, frequency and duration in activities related to housework, yard work/gardening, caregiving, recreation and exercise, during week before interview	**YPAS (MET-min) - accelerometer** (see table below)

YPAS (MET-min) - accelerometer

YPAS	Acc	men ρ	men P	women ρ	women P
work	time moderate	0.31	0.0432	-0.17	n.s.
work	continuous time	0.35	0.0237	-0.19	n.s.
exercise	time moderate	0.40	0.0083	0.29	0.0213
exercise	time high	0.39	0.009	0.27	0.0249
exercise	continuous time	0.34	0.0267	0.25	0.01
exercise	total counts	0.54	0.0002	0.28	0.0261
recreation	time moderate	0.25	n.s	-0.26	0.0446
YPAS	TTL time moderate	0.42	0.0059	-0.005	n.s.
YPAS	TTL continuous time	0.42	0.0058	-0.29	0.0233
YPAS	TTL total counts	0.54	0.0002	0.13	n.s.

*YPAS TTL: total weekly energy expenditure

Additional File 1. Summary of correlations of physical activity data assessed by questionnaires and accelerometers with respect to Gender (continued)

Study	*Journal*	*No. of Participants*	*Accelerometer used*	*Accelerometer wear method*	*Questionnaire used in investigation*	*Results*
**Johnson-Kozlow, 2006**	Intl Journal of Behavioral Nutrition and Physical Activity. 2006. 3:7	159 women (Breast Cancer from Women's Healthy Eating and Living Study); Avg, 57 yrs.	MTI Actigraph accelerometer (7164 model) Fort Walton Beach, FL	In a pouch on a belt around the waist midway between the navel and the right side of the body. Wear during waking hours, excepting water activities. Recorded in 1-minute intervals.	7-Day Physical Activity Recall (PAR):<br>Cues and prompts to help participants recall. Rated activity as moderate, hard, or very hard.<br><br>IPAQ:<br>Long form administered by phone. Moderate and vigorous activity in 4 domains (work, home, recreation, transport)	Validity Spearman correlation coefficient of total physical activity scores<br>ρ= 0.73 (PAR)<br>ρ= 0.33 (IPAQ)
**Rosenberg, 2008**	J Phys Act Health. 2008; 5 Suppl 1:S30-44.	n=200, 3 countries, convenience sample (staff, students, affiliated with universities)	Computer Science and Application's Inc. (Shalimar, FL) accelerometer (CSA model 7164)	7 days, during waking hours, min 600 minutes, min 5 valid days, min 1 weekend day, 1min epochs	Long and short IPAQ (sitting items), number of hours and minutes per day, weekday and weekend day, long form: time spent sitting in vehicle was assessed separately	Spearman correlation<br>**Acccounts (<100/min) - total sitting**<br>long form / short form<br>ρ= 0.33 r= 0.34 (total)<br>ρ= 0.26 r= 0.24 (men)<br>ρ= 0.40 r= 0.43 (women)
**Orsini, 2008**	Eur J Epidemiol (2008). 23: 661-667	116 women, 56-75 years from Swedish Mammo-graphy Cohort	MTI Actigraph model 7164	7 consecutive days during waking hours Min. wear =10hr/day, min. days = 4	Short self-administered questionnaire used to assess average physical activity/inactivity during previous year in the Cohort.	Concordance Correlations for validity of total physical activity score:<br>Acc to questionnaire: 0.36 (0.21-0.52)<br>De-attenuated Acc to questionnaire: 0.38 (0.22 - 0.54)
**Wolin, 2008**	Br J Sports Med. 2010 Aug; 44(10):741-6. Epub 2008 Nov 3.	n= 142, 24-70 years, Black, African American, no restriction to usual physical activity	Actical activity monitor (Mini Mitter Co., Bend, OR)	6 consecutive days, on hip, clip, or waistband, during waking hours, ≥4 days, ≥ 10h/d,	IPAQ short form, past 7 days	Spearman correlation<br>~ 1-min bout length<br>**IPAQ-SF - Acc Counts**:<br>ρ= 0.36 P <0.001<br>ρ= 0.58 P <0.001 men<br>ρ= 0.21 P= 0.05 women<br>Agreement between both. k= 0.21, 95%CI: -0.4-0.47

Additional File 1. Summary of correlations of physical activity data assessed by questionnaires and accelerometers with respect to Gender (continued)

Study	*Journal*	*No. of Participants*	*Accelerometer used*	*Accelerometer wear method*	*Questionnaire used in investigation*	*Results*
**Wolin, 2008 continued**	Br J Sports Med. 2010 Aug; 44(10):741-6. Epub 2008 Nov 3.	n= 142, 24-70 years, Black, African American, no restriction to usual physical activity	Actical activity monitor (Mini Mitter Co., Bend, OR)	6 consecutive days, on hip, with clip, or waistband, during waking hours, ≥4 days, ≥ 10h/d, within 10h one 2h period of non-wear time was allowed	IPAQ short form, past 7 days	~ 10-min bout length<br>**IPAQ-SF - Acc Counts:**<br>ρ= 0.26 P= 0.002<br>ρ= 0.48 P= 0.003 men<br>ρ= 0.07 P= 0.48 women<br>Agreement between both: k= 0.04, 95% CI: 0.01-0.06.
**Hagiwara, 2008**	Geriatr Gerontol Int. 2008 Sep; 8(3):143-51.	n= 325, 65+ years, no cognitive disorder or deficiency in activities of daily living	Life Corder; Suzuken, Tokyo, Japan	3 days, after filling out PA questionnaire, during waking hours	PASE (physical activity scale for the elderly), leisure time, household, work-related, past 7 days	Spearman rank correlation<br>**PASE-Acc steps**:<br>total: ρ= 0.17 (P= 0.01)<br>men: ρ= 0.38 p <0,001<br>women: ρ= -0.02<br>**PASE-Acc Energy expenditure**:<br>total: ρ= 0.16 P= 0.02<br>men: ρ= 0.35 p= 0,001<br>women: ρ= 0.01
**Jacobi, 2009**	Eur J Epidemiol. 2009; 24(4):171-9. Epub 2009 Mar 13.	n= 141 (160), 18-74 years, low habitual PA levels	ActiGraph (model 7164, Manufacturing Technology, Inc., FL, USA)	7 consecutive days, 1 min epochs, worn on nylon pouch, on a belt, around waist, during all waking hours, min 4 days, min 8h/d	Modifiable AQ (MAQ) French version, past year PA leisure time & work, as well as sedentary behavior, all non-occupational non-leisure PA was added to the original Qu.	Spearman correlations<br>**women:**<br>**total PA (counts/d) - total leisure time PA:**<br>ρ= 0.25 (P <0.05) (h/week), ρ= 0.18 (METh/week)<br>**total PA (counts/d) - moderate intensity leisure time PA:**<br>ρ= 0.20 (P<0.1) (h/week), ρ= 0.22 (P <0.05) (METh/week)<br>**total PA (counts/d) - vigorous intensity leisure time PA:**<br>ρ= 0.08 (h/week), ρ= 0.08 (METh/week)<br>**total PA (counts/d) vs. total activity (METh/week)**<br>ρ= 0.07<br>**men:**<br>**total PA (counts/d) - total leisure time PA:**<br>ρ= 0.04 (h/week), ρ= -0.05 (METh/week)<br>**total PA (counts/d) - moderate intensity leisure time PA**:<br>ρ= 0.04 (h/week), ρ= 0.03 (METh/week)<br>**total PA (counts/d) - vigorous intensity leisure time PA:**<br>ρ= -0.15 (h/week), ρ= -0.15 (METh/week)

Additional File 1. Summary of correlations of physical activity data assessed by questionnaires and accelerometers with respect to Gender (continued)

Study	*Journal*	*No. of Participants*	*Accelerometer used*	*Accelerometer wear method*	*Questionnaire used in investigation*	*Results*
**Jacobi, 2009 continued**	Eur J Epidemiol. 2009; 24(4):171-9. Epub 2009 Mar 13.	n= 141 (160), 18-74 years, low habitual PA levels	ActiGraph (model 7164, Manufacturing Technology, Inc., FL, USA)	7 consecutive days, 1 min epochs, worn on nylon pouch, on a belt, around waist, during all waking hours, min 4 days, min 8h/d	Modifiable AQ (MAQ) French version, past year PA leisure time & work, as well as sedentary behavior, all non-occupational non-leisure PA was added to the original Qu.	**total PA (counts/d) vs. total activity (METh/week)**<br>ρ= 0.39 P<0.01<br>**all:**<br>**total PA (counts/d) - total leisure time PA:**<br>ρ= 0.19 (P <0.05) (h/week), ρ= 0.09 (METh/week)<br>**total PA (counts/d) - moderate intensity leisure time PA:**<br>ρ= 0.14 (P<0.1) (h/week), ρ= 0.14 (P <0.1) (METh/week)<br>**total PA (counts/d) - vigorous intensity leisure time PA:**<br>ρ= -0.02 (h/week), ρ= -0.02 (METh/week)<br>**total PA (counts/d) vs. total activity (METh/week)**<br>ρ= 0.18 P<0.05
**Li, 2009**	Eur J Clin Nutr. 2009 Dec; 63(12):1448-51. Epub 2009 Jul 29.	n= 369, 65 +- 6 non-diabetic subgroup of Malmö Diet and Cancer cohort	Computer Science and Application's Inc. (Shalimar, FL) accelerometer (CSA model 7164)	4 consecutive days, during waking hours, total no. of activity counts categorized as gender specific quintiles: low, quintile 1; moderate, quintile 2-4; high, quintile 5	Leisure time comprehensive questionnaire (MDC-score), 18 questions range of 4 seasons (past year), simple leisure time questionnaire (past year), categorized as low(sedentary), moderate (light exercise >=4h/week), high (regular exercise >=3h/week)	Spearman correlation<br>**MDC-score - CSA:**<br>men: ρ= 0.35 P<0.01<br>women: r= 0.24 P<0.01<br>**Simple-score-CSA**:<br>men: ρ= 0.32 P<0.01<br>women: ρ= 0.15 P<0.05
**Emaus, 2010**	Scand J Public Health. 2010 Nov; 38(5 Suppl):105-18.	n= 270 (138 women, 132 men), 30-87 years	ActiGraph GT1M	7 consecutive days during waking hours, on elastic belt on right hip, start time programmed to 07.00 am, Min. 10 hours/d, 15s epochs, integrated to 1 minute for data analysis	Questionnaires used in Tromso survey, leisure activity and work activity	Spearman correlation<br>**Correlation Qu (leisure activity) vs. Acc activity:**<br>women<br>ρ= 0.23 , p<0.05 (overall activity), ρ= -0.22, p<0.05 (light PA),<br>ρ= 0.28, p<0.01 (MVPA), ρ= 0.27, p<0.01 (vigorous PA)<br>men<br>ρ= 0.23 , p<0.01 (overall activity), ρ= -0.23, p<0.05 (light PA),<br>ρ= 0.25, p<0.01 (MVPA), ρ= 0.29, p<0.01 (vigorous PA)

Additional File 1. Summary of correlations of physical activity data assessed by questionnaires and accelerometers with respect to Gender (continued)

Study	*Journal*	*No. of Participants*	*Accelerometer used*	*Accelerometer wear method*	*Questionnaire used in investigation*	*Results*
**Emaus, 2010 continued**	Scand J Public Health. 2010 Nov; 38(5 Suppl):105-18.	n= 270 (138 women, 132 men), 30-87 years	ActiGraph GT1M	7 consecutive days during waking hours, on elastic belt on right hip, start time programmed to 07.00 am, Min. 10 hours/d, 15s epochs, integrated to 1 minute for data analysis	Questionnaires used in Tromso survey, leisure activity and work activity	**Correlation Qu PA (work) vs. AccPA:**<br>women<br>ρ= 0.11 (overall activity), ρ= 0.40, $p<0.001$ (light PA), ρ= 0.05 (MVPA), ρ= -0.13 (vigorous PA)<br>men<br>ρ= 0.08 (overall activity), ρ= 0.29, $p<0.01$ (light PA), ρ= 0.04 (MVPA), ρ= -0.15 (vigorous PA)
**Rosenberg, 2010**	Journal of Physical Activity and Health, 2010, 7, 697-705	n= 654 (accelerometer) (300 women, 354 men	ActiGraph accelerometer (model WAM 7164)	7 days, during waking hours, attached on nylon belt around waist, on right hip, 1 min epochs, 1 valid hour -> consecutive minutes of zero counts ≤ 30 minutes, min 3 days, min 10 hours per day	The Sedentary Behavior Questionnaire (SBQ), assesses amount of time spent: watching TV, playing computer/video games, sitting while listening to music, sitting and talking on the phone, doing paperwork or office work, sitting and reading, playing a musical instrument, doing arts and crafts, sitting and driving/riding in a car, bus or train), completely separate for weekdays and weekend days	Partial correlations for overweight samples<br><br>**SBQ score vs. Accelerometer total activity mins/day**<br>r= -0.03 men (total hours/week)<br>r= 0.10 women (total hours/week)<br><br>**SBQ score vs. Accelerometer minutes with counts < 100**<br>r= -0.01 men (total hours/week)<br>r= 0.10 women (total hours/week)

Additional File 1. Summary of correlations of physical activity data assessed by questionnaires and accelerometers with respect to Gender (continued)

Study	*Journal*	*No. of Participants*	*Accelerometer used*	*Accelerometer wear method*	*Questionnaire used in investigation*	*Results*
**Evenson, 2010**	Intl Journal of Behavioral Nutrition and Physical Activity. 2010. 7:21	177 pregnant women (for validity assessment), n=120 with complete Actigraph data	MTI Actigraph accelerometer (7164 model) Fort Walton Beach, FL	waking hours for 7 days. Remove for sleeping, bathing, and swimming, 1 minute epochs, complete data with non-missing counts over 70% of a standard measurement day for all 7 days.	PIN3 Physical Activity Questionnaire. One-week recall. Interviewer administered, capturing moderate and vigorous physical activity<br><br>PIN3 Diary Card used to assess concurrent related validity of the questionnaire. Filled out on a daily basis to be mailed back after the week.	**Total physical activity in MET-hours/week- total counts/week:**<br>Complete Actigraph sample (120): 0.31 (0.13 - 0.49)<br>**Moderate PA hours/week (Qu) - hours/week (Acc)**<br>3-6 METs<br>0.07 (-0.11, 0.24) Freedson et. al*<br>0.24 (0.05, 0.43) Schwartz et. al*<br>0.06 (-0.12, 0.24) Troiano et. al*<br>4.8-7.1 METs<br>0.05 (-0.14, 0.24) Freedson et. al*<br>0.05 (-0.13, 0.23) Schwartz et. al*<br>0.04 (-0.15, 0.22) Troiano et. al*<br>**Vigorous PA hours/week (Qu) - hours/week (Acc)**<br>3-6 METs<br>0.38 (0.20, 0.56) Freedson et. al*<br>0.44 (0.28, 0.60) Schwartz et. al*<br>0.40 (0.22, 0.57) Troiano et. al*<br>4.8-7.1 METs<br>0.32 (0.14, 0.51) Freedson et. al*<br>0.32 (0.14, 0.50) Schwartz et. al*<br>0.34 (0.15, 0.52) Troiano et. al*<br>**MVPA hours/week (Qu) - hours/week (Acc)**<br>3-6 METs<br>0.17 (0.004, 0.35) Freedson et. al*<br>0.32 (0.14, 0.50) Schwartz et. al*<br>0.16 (-0.01, 0.34) Troiano et. al*<br>0.32 (0.14, 0.50) total counts/week<br>4.8-7.1 METs<br>0.20 (0.02, 0.38) Freedson et. al*<br>0.21 (0.04, 0.38 Schwartz et. al*<br>0.19 (0.01, 0.37) Troiano et. al*<br>0.21 (0.04, 0.38) total counts/week<br>*Freedson et. al: 1952-5724 (Moderate); > = 5725 (Vigorous)<br>Swartz et al: 574-4944 (Moderate); > = 4945 (Vigorous)<br>Troiano et al: 2020-5998 (Moderate); > = 5999 (Vigorous)

Additional File 1. Summary of correlations of physical activity data assessed by questionnaires and accelerometers with respect to Gender (continued)

Study	*Journal*	*No. of Participants*	*Accelerometer used*	*Accelerometer wear method*	*Questionnaire used in investigation*	*Results*
**Hagstromer et al., 2010**	J Phys Act Health. 2010 Jul;7(4):541-50	N=980, 18-65 years	Actigraph MT1 model 7164 (Manufacturing Technology Inc, Fort Walton Beach, FL, USA)	7 consecutive days, at least 4 days with at least 10 hours, at least 1 weekend day	IPAQ long form	Spearman's Rank correlation coefficient between IPAQ and accelerometer<br>**Moderate activity ρ= 0.27, P<0.01**<br>Men: ρ=0.32, P<0.05<br>Women: ρ=0.22, P<0.01<br>**Vigorous activity ρ=0.31, P<0.01**<br>Men: ρ=0.31, P<0.01<br>Women: ρ=0.29, P<0.01<br>**Total activity ρ=0.28, P<0.01**<br>Men: ρ=0.30, P<0.01<br>Women: ρ=0.27, P<0.01
**Lee, 2011**	Int J Behav Nutr Phys Act. 2011 Aug 1; 8:81.	n= 1270 (42.9 ± 14.4 years)	ActiGraph GT1M	4 consecutive days, around waist, 2 weekdays+2 weekend days, during waking hours, first day always Thursday, Friday, Saturday), less than 600 min of registered time/day-> invalid, 1 min epoch,	IPAQ-C, short form (9items vs. 31items long form), equivalent psychometric properties to the long form	Spearman correlation<br>**IPAQ-C (moderate PA) vs. Acc (min in moderate PA)**<br>ρ= 0.10, P<0.05 men; ρ= 0.09, P<0.05 women<br>ρ= 0.05 ≤29 years; ρ= 0.09 30-49 years; ρ= P<0.05 ≥50 years<br>ρ= 0.10 BMI ≥25; ρ= 0.09, P<0.01 BMI < 25<br>**IPAQ-C (vigorous PA) vs. Acc (min in vigorous PA)**<br>ρ= 0.23, P<0.001 men; ρ= 0.09, P<0.05 women<br>ρ= 0.21, P<0.001 ≤29 years; ρ= 0.12, P<0.01 30-49 years;<br>ρ= P<0.01 ≥50 years<br>ρ= 0.22, P<0.001 BMI ≥25; ρ= 0.14, P<0.001 BMI < 25<br>**IPAQ-C (total MET) vs. Acc (counts/h)**<br>ρ= 0.18, P<0.001 men; ρ= 0.15, P<0.001 women<br>ρ= 0.04 ≤29 years; ρ= 0.19, P<0.001 30-49 years;<br>ρ= 0.25, P<0.001 ≥50 years<br>ρ= 0.21, P<0.001 BMI ≥25; ρ= 0.14, P<0.001 BMI < 25

Additional File 1. Summary of correlations of physical activity data assessed by questionnaires and accelerometers with respect to Gender (continued)

Study	*Journal*	*No. of Participants*	*Accelerometer used*	*Accelerometer wear method*	*Questionnaire used in investigation*	*Results*
**Tomioka, 2011**	J Epidemiol. 2011 Nov 5; 21(6):459-65. Epub 2011 Sep 24	325 (164 men, 161 women), >=65 years,	Kenz Lifecorder PLUS, Suzuken Co., Ltd., Nagoya, Japan	Left or right hip, on waist belt, ≥ 2 weeks (preferably 4 weeks), starting on day after 1st IPAQ, 2nd IPAQ on 14th day of accelerometer wear, during waking hours	IPAQ short form, twice, 2 weeks apart, 2 different sets of MET values: 1. original IPAQ values, 2. modified for use with elderly	Spearman correlation<br>**Acc (total PA in METmin/week) vs. IPAQ (total PA in METmin/week)**<br>65-74: ρ= 0.42 men, ρ=0.49 women<br>75-89: ρ= 0.53 men, ρ= 0.49 women *all P< 0.01*<br>**Acc (total PA in METmin/week) vs. IPAQ (vigorous PA min/week)**<br>65-74: ρ= 0.25 men, P< 0.05, ρ= 0.12 women<br>75-89: ρ= 0.17 men, ρ= 0.17 women<br>**Acc (total PA in METmin/week) vs. IPAQ (moderate PA min/week)**<br>65-74: ρ= 0.26 men, P< 0.05, ρ= 0.13 women<br>75-89: ρ= 0.05 men, ρ= 0.03 women
**Nang, 2011**	BMC Med Res Methodol. 2011 Oct 13;11:141	152 adults (21+ years)	Actical physical activity monitor (Mini Mitter Co., Inc., Bend, OR)	5 consecutive days, right hip with elastic belts, 3 weekdays, 2 weekend days, 15sec epochs, converted to 1min epochs for analysis of energy expenditure, accelerometer data considered valid if: ≥10 hours/d, 5 days	IPAQ, 120 participants before measurement and immediately afterwards, SP2PAQ (Singapore Prospective Study Program PAQ), 43 the other way around	Spearman correlation<br>**IPAQ-Acc:**<br>r= 0.13/ 0.15 corrected (moderate activity),<br>r= 0.18/ 0.31 corrected (vigorous activity), p< 0.05,<br>r= 0.19 (combined)<br>age ≤ 40:<br>0.08/ 0.09 corrected (moderate),<br>0.30/ 0.52 corrected (vigorous), p<0.05<br>age >40:<br>r= 0.21/ 0.24 corrected (moderate),<br>r= -0.07/ -0.01 corrected (vigorous)<br>male:<br>r= 0.24/ 0.27 corrected (moderate),<br>r= 0.28/ 0.48 corrected (vigorous)<br>female:<br>r= 0.12/ 0.13 corrected (moderate),<br>r= 0.05/ 0.09 corrected (vigorous)<br>**SP2PAQ-Acc**:<br>r= 0.24/ 0.27 corrected (moderate activity),p< 0.05,<br>r= 0.42, p< 0.0001/ 0.73, p< 0.05 corrected (vigorous activity), p< 0.05, r= 0.19 (combined)<br>age ≤ 40:<br>r= 0.21/ 0.24 corrected, p< 0.05 (moderate)

Additional File 1. Summary of correlations of physical activity data assessed by questionnaires and accelerometers with respect to Gender (continued)

Study	*Journal*	*No. of Participants*	*Accelerometer used*	*Accelerometer wear method*	*Questionnaire used in investigation*	*Results*
**Nang, 2011**	BMC Med Res Methodol. 2011 Oct 13;11:141	152 adults (21+ years)	Actical physical activity monitor (Mini Mitter Co., Inc., Bend, OR)	5 consecutive days, right hip with elastic belts, 3 weekdays, 2 weekend days, 15sec epochs, converted to 1min epochs for analysis of energy expenditure, accelerometer data considered valid if: ≥10 hours/d, 5 days	IPAQ, 120 participants before measurement and immediately afterwards, SP2PAQ (Singapore Prospective Study Program PAQ), 43 the other way around	r= 0.48, p< 0.0001/ 0.83 corrected (vigorous), p<0.05<br>age >40:<br>r= 0.27/ 0.30 corrected (moderate);<br>r= 0.48, p< 0.0015/ 0.85 corrected (vigorous)<br>male:<br>r= 0.16/ 0.18 corrected (moderate) p< 0.05,<br>r= 0.34/ 0.59 corrected (vigorous) p< 0.05<br>female:<br>0.29/ 0.33 corrected (moderate),<br>0.05/ 0.09 corrected (vigorous)<br>corrected: for within-person variation in acc measurement
**Lee, 2011**	J Community Health; 26 April 2011	262 African American women, 148 Hispanic or Latina women from Health Is Power study	Uni-directional ActiGraph GT1M accelerometer Pensacola, FL	Wear for 7 days at all times except for in the shower or sleeping – included a log to record times put on and taken off each day.	IPAQ – interviewer- administered baseline health assessment for the last 7 days. MET-minutes per week<br><br>CALQ (Check And Line Questionnaire) – self reported for 7 days. Measures number of 15-minute physical activity sessions and determines continuity and intensity	Spearman's Rho:<br>**IPAQ work:** 0.078<br>**IPAQ transportation:** 0.109<br>**IPAQ domestic:** -0.026<br>**IPAQ leisure:** 0.026<br>**IPAQ walking:** 0.077<br>**IPAQ moderate:** -0.022<br>**IPAQ vigorous:** 0.037<br>**IPAQ total PA**: 0.059<br>[IPAQ and CALQ were significantly correlated only for transportation (0.118), leisure (0.119), and vigorous (0.115)]<br>Pearson's r:<br>CALQ to Accelerometer: 0.186 (p<0.05)

Additional File 1. Summary of correlations of physical activity data assessed by questionnaires and accelerometers with respect to Gender (continued)

Study	*Journal*	*No. of Participants*	*Accelerometer used*	*Accelerometer wear method*	*Questionnaire used in investigation*	*Results*
**Grimm, 2012**	J Aging Phys Act. 2012 Jan;20(1):64-79	n= 127, 50-87 years, self-reported being healthy, no limitations for engaging in physical activities	The ActiGraph 7164 (ActiGraph, LLC Pensacola, FL)	7 consecutive days, on elastic belt, over right hip, during all waking hours (except water activities)	IPAQ	Spearman rank-order correlations **IPAQ (min/day) - Acc (min/day)**<br>**accumulated Acc minutes**<br>IPAQ / Acc / Men / women / all<br>sitting / sedentary / 0.404* / 0.336** / 0.357**<br>moderate / moderate lifestyle / 0.288 / 0.137 / 0.164<br>walking / moderate walking / 0.262 / 0.316** / 0.294**<br>vigorous / vigorous / 0.217 / 0.153 / 0.176<br>total PA / MVPA / 0.440** / 0.180 / 0.237**<br><br>**bout Acc minutes**<br>IPAQ / Acc / Men / women / all<br>sitting / sedentary<br>moderate / moderate lifestyle / 0.051 / 0.050 / 0.042<br>walking / moderate walking / 0.138 / 0.391** / 0.276**<br>vigorous / vigorous / 0.175 / 0.131 / 0.166<br>total PA / MVPA / 0.275 / 0.126 / 0.169<br>*P<0.05, **P< 0.01
**Kwak, 2012**	J Phys Act Health. 2012 Nov;9(8):1130-7. Epub 2011 Dec 27.	N=440, mean age=49.4 years, 44% males	ActiGraph GT1M (ActiGraph, Pensacola, Florida, USA)	7 days, during waking hours, attached to the center of gravity	International Physical Activity Questionnaire (IPAQ) long form, at least 4 days with at least 10 hours/day	Spearman correlation<br>IPAQ-L work vs. accelerometer (min/day)<br>**Total**<br>Accelerometer-MVPA-work: ρ=0.46, P<0.01<br>Accelerometer-work: ρ= 0.39, P<0.01<br>Accelerometer-total: ρ= 0.26, P<0.01<br>**Men**<br>Accelerometer-MVPA-work: ρ=0.44, P<0.01<br>Accelerometer-work: ρ= 0.33, P<0.01<br>Accelerometer-total: ρ= 0.21, P<0.01<br>**Women**<br>Accelerometer-MVPA-work: ρ=0.49, P<0.01<br>Accelerometer-work: ρ= 0.41, P<0.01<br>Accelerometer-total: ρ= 0.36, P<0.01<br>**BMI**<br>(<25kg/m²) Accelerometer-MVPA-work: ρ=0.44, P<0.01<br>Accelerometer-work: ρ=0.41, P<0.01<br>Accelerometer-total: ρ=0.36, P<0.01

Additional File 1. Summary of correlations of physical activity data assessed by questionnaires and accelerometers with respect to Gender (continued)

Study	*Journal*	*No. of Participants*	*Accelerometer used*	*Accelerometer wear method*	*Questionnaire used in investigation*	*Results*
**Kwak, 2012 (continued)**	J Phys Act Health. 2012 Nov;9(8):1130-7. Epub 2011 Dec 27.	N=440, mean age=49.4 years, 44% males	ActiGraph GT1M (ActiGraph, Pensacola, Florida, USA)	7 days, during waking hours, attached to the center of gravity	International Physical Activity Questionnaire (IPAQ) long form, at least 4 days with at least 10 hours/day	(25-30 kg/m²) Accelerometer-work: ρ= 0.55, P<0.01<br>Accelerometer-work: ρ=0.43, P<0.01<br>Accelerometer-total: ρ=0.28, P<0.01<br>(≥30kg/m²) Accelerometer-total: ρ= 0.27, P<0.01<br>Accelerometer-work: ρ=0.26, n.s.<br>Accelerometer-total: ρ=0.01, n.s.
**Mâsse, 2012**	J Phys Act Health. 2012 Feb;9(2):237-48	Women, n= 130 African American, n= 130 Hispanic, 40-70 years, not suffering from any health conditions that would preclude them being active	CSA model 7164 (Computer Science Applications, Inc.)	7 days, over right hip, during all waking hours (except water activities)	The Checklist questionnaire, 64 items, household (24), yard (10), family (6), church/volunteer work (11), transportation (2), self-administered and partially interviewer-administered, assesses frequency and duration of PA, based on Minnesota Leisure Time PAQ<br>The Global questionnaire, 8 items, interviewer-administered, assesses moderate to vigorous intensity activities for 6 domains of PA (occupation, household, yard, family, church/volunteer work, transportation)	**Checklist questionnaire - Accelerometer**<br>METmin/day of moderate to vigorous intensity activity<br>- total accelerometer counts: r= 0.30, P< 0.05<br>- total min ≥3METs (accelerometer): r= 0.23, P< 0.05<br><br>**Global questionnaire - Accelerometer**<br>METmin/day of moderate to vigorous intensity activity<br>- total accelerometer counts: r= 0.23, P< 0.05<br>- total min ≥3METs (accelerometer): r= 0.22, P< 0.05
**Sullivan 2012**	Int J Behav Nutr Phys Act. 2012; 9: 13.	n= 157 Indians, factory workers and co-residence spouses	Actigraph 7164	At least 4 full days during waking hours, min 10 hours per day, 1min epochs, on right hip (except water activities)	IMS-PAQ (Indian Migration Study), predominantly open questions, last one month	Spearman rank correlations IMS-PAQ – accelerometer<br>Total sample: ρ= 0.41 P< 0.001 (total activity)<br>ρ= 0.17 P< 0.05 (light activity)<br>ρ= 0.47 P< 0.001 (MVPA)<br>Men: ρ= 0.40 P< 0.001 (total activity)<br>ρ= 0.08 (light activity)<br>ρ= 0.37 P< 0.001 (MVPA)<br>Women: ρ= 0.28 (total activity)<br>ρ= 0.45 P< 0.05 (light activity)<br>ρ= -0.01 (MVPA)

Additional File 1. Summary of correlations of physical activity data assessed by questionnaires and accelerometers with respect to Gender (continued)

Study	*Journal*	*No. of Participants*	*Accelerometer used*	*Accelerometer wear method*	*Questionnaire used in investigation*	*Results*
**Dahl-Petersen, 2013**	Med Sci Sports Exerc. 2013 Apr;45(4):728-36	N=1508, ≥18 years, Inuit in Greenland	Actiheart (CamNtech Ltd, Cambridge, UK)	4 days, at least 2 days of valid data	IPAQ long form	Spearman correlation coefficient between IPAQ and accelerometer<br>**Total physical activity**<br>Men: 0.33, P<0.001<br>Women: 0.28, P<0.001<br>**Moderate intensity**<br>Men: 0.25, P<0.001<br>Women: 0.19, P<0.001<br>**Vigorous intensity**<br>Men: 0.27, P<0.001<br>Women: 0.17, P<0.001
**Segura-Jiménez, 2013**	Clin Exp Rheumatol. 2013 Nov-Dec;31(6 Suppl 79):S94-101. Epub 2013 Dec 2.	N=123 spanish women with fibromyalgia	SenseWear Pro$_3$ Armband (Body Media, Pittsburgh, PA)	Worn on right upper arm over triceps, 9 consecutive days, 1-min intervals, first and last days excluded to minimize reactivity, at least 7 days,	Modified International Physical Activity Questionnaire (IPAQ), 2 items added about time spent lying	Pearson correlation coefficient IPAQ-accelerometer<br>r= -0.12 P= 0.18 (sitting)<br>r= 0.06 P= 0.54 (moderate PA)<br>r= 0.04 P= 0.63 (vigorous PA)<br>r=0.11 P= 0.21 (total PA)
**Gabriel, 2013**	Menopause. Feb 2013; 20(2): 152–161.	N=148 women, 42-50 years,	ActiGraph GT1M (Pensacola, Florida)	On dominant hip, 7 consecutive days, 24 hours, at least 10 hours/day	Modifiable Activity Questionnaire (MAQ)	Pearson correlation coefficient questionnaire-accelerometer<br>r= 0.39, P<0.001 (total PA)<br>r= 0.07, n.s.<br>r=0.38, P<0.001<br>r=0.42, P<0.001

Additional File 1. Summary of correlations of physical activity data assessed by questionnaires and accelerometers with respect to Gender (continued)

Study	*Journal*	*No. of Participants*	*Accelerometer used*	*Accelerometer wear method*	*Questionnaire used in investigation*	*Results*
**Sabia, 2014**	Am J Epidemiol. 2014 Mar 15;179(6):781-90.	n= 3,975, 60-83 years	GeneActiv; Activinsights Ldt., Cambs, UK)	Wrist-worn, on non-dominant wrist, 9 consecutive days, 24 hours, at least 2 weekdays and 2 weekend days for at least 16 hours per day	Modified version of the Minnesota Leisure Time Physical Activity Questionnaire, 20 items on amount of time spent in: walking, sports, gardening, housework, do-it-yourself activity (building, repairing,..), 2 open-ended questions on "other activities"	Spearman correlation questionnaire-accelerometer (total PA)<br><br>Total: ρ=0.33 (95% CI: 0.30-0.36)<br>Women: ρ= 0.32 (95% CI: 0.26-0.37)<br>Men: ρ= 0.33 (95% CI: 0.30-0.37)<br>60-65 years: ρ=0.30 (95% CI: 0.25-0.35)<br>66-70 years: ρ=0.36 (95% CI: 0.31-0.41)<br>71-75 years: ρ=0.34 (95% CI: 0.27-0.40)<br>76-83 years: ρ=0.26 (95% CI: 0.20-0.33)

Abbreviations: IPAQ, International Physical Activity Questionnaire; Acc, accelerometry; h, hours; d, days; BWHS, Black Women's Health Study; Qu, Questionnaire; PYTPYQ, past year total physical activity questionnaire; PA, physical activity; PAQ-EJ, physical activity questionnaire for elderly Japanese; MET, metabolic equivalent of task; PASE, physical activity scale for the elderly; EPIC, European Prospective Investigation into Cancer and Nutrition; EE, energy expenditure; GPAQ, Global Physical Activity Questionnaire; MVPA, moderate-vigorous physical activity; SP2PAQ, Singapore Prospective Study Program Physical Activity Questionnaire; IMS, Indian Migration Study
