# Supplementary material for: Accelerometry and physical activity questionnaires - a systematic review
Source: BMC Public Health. 2016 Jun 16;16:515. doi: 10.1186/s12889-016-3172-0 (PMC4910242; doi:10.1186/s12889-016-3172-0)
Supplement: Additional file 2: — Summary of correlations of physical activity data assessed by questionnaires and accelerometers with respect to BMI. (PDF 183 kb) [file 12889_2016_3172_MOESM2_ESM.pdf]

Additional File 2. Summary of correlations of physical activity data assessed by questionnaires and accelerometers with respect to BMI

Study	***Journal***	***No. of Participants***	***Accelerometer used***	***Accelerometer wear method***	***Questionnaire used in investigation***	***Results***
**Timperio, 2003**	J Sci Med Sport. 2003 Dec; 6(4):477-91.	n= 144<br>n= 122 (for validation data)<br>18+ years	MTI/CSA accelerometer (model 7164)	7 consecutive days, during waking hours, attached with adjustable belt, on right hip, 1 minute epochs	One week recall PAQ, assesses frequency and duration of walking, other moderate intensity activity and vigorous intensity activity, questionnaire administered twice -> 3 days apart, again after accelerometer measurement	Spearman correlation<br>**Correlation PAQ (min/day) vs. Accelerometer (min/day)**<br>PAQ (duration of ≥3.0 METs) vs. Acc:<br>men women<br>overall ρ= 0.29 p <0.05 ρ= 0.25 p <0.05<br>BMI ≤25 ρ= 0.26 ρ= 0.39 p<0.01<br>BMI >25 ρ= 0.36 p<0.05 ρ= 0.09<br>PAQ (duration of 3.0-5.9 METs) vs. Acc:<br>men women<br>overall ρ= 0.40 p <0.01 ρ= 0.19<br>BMI ≤25 ρ= 0.37 p<0.05 ρ= 0.19<br>BMI >25 ρ= 0.39 p<0.05 ρ= 0.24<br>PAQ (duration of 6.0+ METs) vs. Acc:<br>men women<br>overall ρ= 0.19 ρ= 0.10<br>BMI ≤25 ρ= -0.06 ρ= 0.52 p≤0.001<br>BMI >25 ρ= 0.40 p<0.05 ρ= -0.36 p<0.05
**Friedenreich 2006**	Am J Epidemiol. 2006 May 15; 163(10):959 -70. Epub 2006 Mar 8	n= 154, 35-65 years, residence in Calgary Health Region of Alberta, Canada	MTI ActiGraph (Manufacturing Technology Inc., Fort Walton Beach, FL, USA)	7 days during waking hours, 4 one week periods within 1 year, 12 weeks apart to cover all seasons during waking hours, min 3 days, min 10 hours/day	PYTPYQ (past year total physical activity qu), occupational, household, recreational activity, at baseline, 9 weeks after baseline, after 12 months	Spearman rank correlation<br>**PYTPAQ - Acc** (total PA)<br>ρ= 0.26 (total population) p <0.05<br>ρ= 0.39 (male) p <0.001<br>ρ= 0.14 (female)<br>ρ= 0.43 (age < 50) p <0.001<br>ρ= 0.05 (age ≥ 50)<br>ρ= 0.38 (BMI<25) p <0.05<br>ρ= 0.19 (BMI ≥ 25)<br>ρ= 0.26 (moderate PA) *significant<br>ρ= 0.34 (vigorous PA) *significant<br>ρ= -0.08 (light PA)

Additional File 2. Summary of correlations of physical activity data assessed by questionnaires and accelerometers with respect to BMI (continued)

Study	***Journal***	***No. of Participants***	***Accelerometer used***	***Accelerometer wear method***	***Questionnaire used in investigation***	***Results***
**Cust, 2008**	Intl Journal of Behavioral Nutrition and Physical Activity. 2008. 5:33	100 men, 82 women, 50-65	ActiGraph (MTI) model 7164, LLC, Fort Walton Beach, FL	Right hip, attached to elastic belt. 3 separate 7-consecutive day periods during follow up each 14 weeks apart. Wear during waking hours except when in water. 1 minute epochs. Valid days min. 10 hrs. weeks with fewer than 4-days valid data were excluded	EPIC questionnaire - for PA in past-year in occupational, leisure, and home domains.<br><br>Friedenreich Lifetime Total Physical Activity Questionnaire (LTPAQ). Frequency, duration, and intensity of physical activity in 4 different domains (work, recreation, home, transport) over lifetime.	**EPIC & Accelerometer:**<br>PA: ρ= 0.29 (0.15 - 0.42)<br>Cambridge Index: ρ= 0.32 (0.19 - 0.45)<br>Occupational Level Index: ρ= 0.37 (0.22 - 0.51)<br>**EPIC Qu measures - total PA (accelerometer)**<br>Total non-occupational: ρ= 0.21 (0.07 - 0.35)<br>Males: ρ= 0.24 (0.05 - 0.42)<br>Females ρ= 0.16 (-0.06 - 0.36)<br><27.2 BMI (89): ρ= 0.33 (0.14 - 0.51)<br>≥27.2 BMI (92): ρ= 0.12 (-0.09 - 0.32)<br><58yrs (95): ρ= 0.25 (0.05 - 0.43)<br>≥58yrs (87): ρ= 0.18 (-0.03 - 0.37)<br>Full Time work (113): ρ= 0.17 (-0.02 - 0.34)<br>Other (68): ρ= 0.30 (0.07 - 0.50)<br>Vigorous Activity (self-rated): ρ= 0.18 (0.04 - 0.50)<br>Vigorous Activity (MET): ρ= 0.23 (0.09 - 0.37)<br>Light-Moderate Activity: ρ= 0.19 (0.05 - 0.33)
**Lee, 2011**	Int J Behav Nutr Phys Act. 2011 Aug 1; 8:81.	n= 1270 (42.9 ± 14.4 years)	ActiGraph GT1M	4 consecutive days, around waist, 2 weekdays+2 weekend days, during waking hours, first day always Thursday, Friday, Saturday), less than 600 min of registered time/day-> invalid, 1 min epoch,	IPAQ-C, short form (9items vs. 31items long form), equivalent psychometric properties to the long form	Spearman correlation<br>**IPAQ-C (moderate PA) vs. Acc (min in moderate PA)**<br>ρ= 0.10, P<0.05 men; ρ= 0.09, P<0.05 women<br>ρ= 0.05 ≤29 years; ρ= 0.09 30-49 years; ρ= P<0.05 ≥50 years<br>ρ= 0.10 BMI ≥25; ρ= 0.09, P<0.01 BMI < 25<br>**IPAQ-C (vigorous PA) vs. Acc (min in vigorous PA)**<br>ρ= 0.23, P<0.001 men; ρ= 0.09, P<0.05 women<br>ρ= 0.21, P<0.001 ≤29 years; ρ= 0.12, P<0.01 30-49 years; ρ= P<0.01 ≥50 years<br>ρ= 0.22, P<0.001 BMI ≥25; ρ= 0.14, P<0.001 BMI < 25<br>**IPAQ-C (total MET) vs. Acc (counts/h)**<br>ρ= 0.18, P<0.001 men; ρ= 0.15, P<0.001 women<br>ρ= 0.04 ≤29 years; ρ= 0.19, P<0.001 30-49 years;<br>ρ= 0.25, P<0.001 ≥50 years<br>ρ= 0.21, P<0.001 BMI ≥25; ρ= 0.14, P<0.001 BMI < 25

Additional File 2. Summary of correlations of physical activity data assessed by questionnaires and accelerometers with respect to BMI (continued)

Study	*Journal*	*No. of Participants*	*Accelerometer used*	*Accelerometer wear method*	*Questionnaire used in investigation*	*Results*
**Kwak, 2012**	J Phys Act Health. 2012 Nov;9(8):1130-7. Epub 2011 Dec 27.	N=440, mean age=49.4 years, 44% males	ActiGraph GT1M (ActiGraph, Pensacola, Florida, USA)	7 days, during waking hours, attached to the center of gravity	International Physical Activity Questionnaire (IPAQ) long form, at least 4 days with at least 10 hours/day	Spearman correlation<br>IPAQ-L work vs. accelerometer (min/day)<br>**Total**<br>Accelerometer-MVPA-work: ρ=0.46, P<0.01<br>Accelerometer-work: ρ= 0.39, P<0.01<br>Accelerometer-total: ρ= 0.26, P<0.01<br>**Men**<br>Accelerometer-MVPA-work: ρ=0.44, P<0.01<br>Accelerometer-work: ρ= 0.33, P<0.01<br>Accelerometer-total: ρ= 0.21, P<0.01<br>**Women**<br>Accelerometer-MVPA-work: ρ=0.49, P<0.01<br>Accelerometer-work: ρ= 0.41, P<0.01<br>Accelerometer-total: ρ= 0.36, P<0.01<br>**BMI**<br>(<25kg/m²) Accelerometer-MVPA-work: ρ=0.44, P<0.01<br>Accelerometer-work: ρ=0.41, P<0.01<br>Accelerometer-total: ρ=0.36, P<0.01<br>(25-30 kg/m²) Accelerometer-work: ρ= 0.55, P<0.01<br>Accelerometer-work: ρ=0.43, P<0.01<br>Accelerometer-total: ρ=0.28, P<0.01<br>(≥30kg/m²) Accelerometer-total: ρ= 0.27, P<0.01<br>Accelerometer-work: ρ=0.26, n.s.<br>Accelerometer-total: ρ=0.01, n.s.

Additional File 2. Summary of correlations of physical activity data assessed by questionnaires and accelerometers with respect to BMI (continued)

Study	***Journal***	***No. of Participants***	***Accelerometer used***	***Accelerometer wear method***	***Questionnaire used in investigation***	***Results***
**Warner, 2013**	Am J Health Behav. Mar 2012; 36(2): 168–178.	N=135	Actical; Phillips/Respironics, Bend, OR USA)	6 consecutive days, on hip, with clip, or waistband, during waking hours, ≥4 days, ≥ 10h/d, within 10h max. 2h period of non-wear time	IPAQ short form	Age and gender adjusted Spearman correlation coefficients<br>IPAQ-S vs. accelerometer<br>1min-bout length :<br>Underweight/normal: ρ= 0.28, P=0.14<br>Overweight: ρ= 0.43, P=0.022<br>Obese: ρ= 0.21, P=0.067<br>10min-bout length:<br>Underweight/normal: ρ= 0.36, P=0.055<br>Overweight: ρ= 0.55, P=0.0026<br>Obese: ρ= 0.14, P=0.24

Abbreviations: IPAQ, International Physical Activity Questionnaire; Acc, accelerometry; BWHS, Black Women’s Health Study; Qu, Questionnaire; PYTPYQ, past year total physical activity questionnaire; PA, physical activity; PAQ-EJ, physical activity questionnaire for elderly Japanese; MET, metabolic equivalent of task; PASE, physical activity scale for the elderly; EPIC, European Prospective Investigation into Cancer and Nutrition; EE, energy expenditure; GPAQ, Global Physical Activity Questionnaire; MVPA, moderate-vigorous physical activity; SP2PAQ, Singapore Prospective Study Program Physical Activity Questionnaire;
