# Supplementary material for: Accelerometry and physical activity questionnaires - a systematic review
Source: BMC Public Health. 2016 Jun 16;16:515. doi: 10.1186/s12889-016-3172-0 (PMC4910242; doi:10.1186/s12889-016-3172-0)
Supplement: Additional file 3: — Summary of correlations of physical activity data assessed by questionnaires and accelerometers with respect to age. (PDF 263 kb) [file 12889_2016_3172_MOESM3_ESM.pdf]

Additional File 3. Summary of correlations of physical activity data assessed by questionnaires and accelerometers with respect to Age

Study	*Journal*	*No. of Participants*	*Accelerometer used*	*Accelerometer wear method*	*Questionnaire used in investigation*	*Results*
**Craig, 2003**	Med Sci Sports Exerc. 2003 Aug;35(8):1381-95.	n= 744 (IPAQ long form), n= 781 (IPAQ short form), 12 countries, 18-65 years	Computer Science and Application's Inc. (Shalimar, FL) accelerometer (model 7164)	7 days, between 2 IPAQ measurements, 1 min epochs, min 5 days, min 600 min/day	IPAQ, short form (9 items) -> estimated time spent sitting per week, long form (31 items) -> estimated total time spent in occupational, transport, household, and leisure related physical activity, total time sitting per week	**Correlation IPAQ (long) - Acc**<br>p= 0.33, 95% CI 0.26-0.39 (pooled)<br>**Correlation IPAQ (short) - Acc**<br>p= 0.30, 95% CI 0.23-0.36 (pooled)
**Friedenreich, 2006**	Am J Epidemiol. 2006 May 15;163(10):959-70. Epub 2006 Mar 8	n= 154, 35-65 years, residence in Calgary Health Region of Alberta, Canada	MTI ActiGraph (Manufacturing Technology Inc., Fort Walton Beach, FL, USA)	7 days during waking hours, 4 1-week periods within 1 year, 12 weeks apart to cover all seasons during waking hours, min 3 days, min 10 hours/day	PYTPYQ (past year total physical activity qu), occupational, household, recreational activity, at baseline, 9 weeks after baseline, after 12 months	Spearman rank<br>**Correlation PYTPAQ - Acc** (total PA)<br>r= 0.43 (age < 50) p <0.001<br>r= 0.05 (age ≥ 50)
**Yasunaga, 2007**	Journal of Aging and Physical Activity, 2007, 15, 398-411	n= 147 Japanese, 65-85 years	Modified Kenz Lifecorder, Suzuken Co., Ltd., Nagoya, Aichi, Japan	1 month, every 4 s, attached to waist belt on either right or left side of body, 1 month before PAQ administration, during waking hours	PAQ-EJ (physical activity questionnaire for elderly Japanese), frequency and duration during a typical week in the preceding month	rank-ordered Spearman correlation<br>**Correlation subtotal PAQ-EJ (lower intensity activities) vs. month averaged record for daily PA <3METs (Acc)**<br>r= 0,28<br>**Correlation PAQ-EJ (total score) vs. month averaged daily step count**<br>r= 0.41<br>**Correlation of PAQ-EJ (higher intensity activities) vs. Activity ≥3 METs (Acc)**<br>r= 0,53
**Hagiwara, 2008**	Geriatr Gerontol Int. 2008 Sep;8(3):143-51.	n= 325, 65+ years, no cognitive disorder or deficiency in activities of daily living	Life Corder; Suzuken, Tokyo, Japan	3 days, after filling out PA questionnaire, during waking hours	PASE (physical activity scale for the elderly), leisure time, household, work-related, past 7 days	Spearman rank correlation<br>**Correlation PASE-Acc steps**:<br>total: r= 0.17 (P= 0.01)<br>men: r= 0.38 p <0,001<br>women: r= -0.02<br>**Correlation PASE-Acc Energy expenditure**:<br>total: r= 0.16 P= 0.02<br>men: r= 0.35 p= 0,001<br>women: r= 0.01

Additional File 3. Summary of correlations of physical activity data assessed by questionnaires and accelerometers with respect to Age *(continued)*

Study	*Journal*	*No. of Participants*	*Accelerometer used*	*Accelerometer wear method*	*Questionnaire used in investigation*	*Results*
**Cust, 2009**	Epidemiology. 2009 May;20(3): 433-41.	n= 177, 50-65 years	ActiGraph (model 7164, Health One Technology, Fort Walton Beach, FL, USA)	3 seperate 7 consecutive day-periods (each 14 weeks apart), on right hip, attached to elastic belt, during waking hours, 1 min epochs, ≥ 20 min of consecutive zero counts = non-wear time, min 4 days, min 10 h/d	EPIC Past-Year Questionnaire (PA, METh/week), assesses occupational, leisure, and household PA, IPAQ (short form, past 7 days)<br>(confidence rating: self-rated confidence in recalling past physical activity)	Correlation<br>**Acc METh/wk - EPIC :**<br>r= 0.30 (0.10-0.48) high confidence (total PA)<br>r= 0.22 (0.02-0.41) low confidence<br>r= 0.22 (0.00-0.43) high confidence (vigorous PA, METh/week)<br>r= 0.11 (-0.10-0.30) low confidence<br>Acc **METh/wk - IPAQ (METh/week)**:<br>r= 0.26 (0.04-0.45) high confidence (total PA)<br>r= 0.27 (0.07-0.46) low confidence<br>r= 0.30 (0.09-0.49) high confidence (moderate+vigorous PA)<br>r= 0.27 (0.07-0.45) low confidence<br>r= 0.35 (0.08-0.55) high confidence (moderate PA)<br>r= 0.01 (-0.17-0.20) low confidence<br>r= 0.28 (0.05-0.49) high confidence (vigorous PA)<br>r= 0.32 (0.13-0.48) low confidence
**Harris, 2009**	Med Sci Sports Exerc. 2009 Jul;41(7):1392-402	n= 234, ≥ 65 years	GT1M (ActiGraph) Yamax Digi-walker SW-200 pedometer (over hip, 7 days)	7 days, over hip on a belt, all day, <5d= exclusion, uniaxial, 30 Hz, 5s epochs	17-item Zuthpen PAQ (EE in kcals), past week, month, usually, added light/heavy household activities bc Zuthpen doesn't ask, added dog walking	**Correlation Zuthpen (modified) - accelerometer activity counts**:<br>r= 0.34, P <0.001<br>**Correlation Zuthpen (traditional)** (without housework included)<br>r= 0.35, P<0.001<br>**Correlation Zuthpen - Acc/pedo. step counts**:<br>r=0.35 and r= 0.36, P<0.001
**Trinh, 2009**	Journal of Physical Activity and Health, 2009, 6(Suppl 1), S46-S53	n= 169 adults, Vietnamese, 25-64 years	ActiGraph model 7164, GT1M	7 consecutive days, during daytime, before the first and last GPAQ assessment, 20 consecutive minutes of zero counts were considered as non-wear time, min 10h/day, min 5 valid days	GPAQ (Global Physical Activity Questionnaire), assesses frequency and duration of PA during work (including household), transportation, and leisure time as well as time spent sitting or reclining during a usual week, twice during dry season (2 weeks apart), twice during wet season (2 weeks apart)	Spearman correlation<br>**Correlation GPAQ - Accelerometer**<br>dry season / wet season<br>r= 0.17 / r= 0.09 (moderate PA)<br>r= -0.03 / r= 0.02 (vigorous PA)<br>r= 0.19 / r= 0.09 (total score vs. MVPA Acc)<br>r= 0.33 / r= 0.19 (total score vs. Total counts)<br>r= 0.22 / r= 0.31 (GPAQ sitting & reclining time vs. Sedentary time)

Additional File 3. Summary of correlations of physical activity data assessed by questionnaires and accelerometers with respect to Age (continued)						
Study	*Journal*	*No. of Participants*	*Accelerometer used*	*Accelerometer wear method*	*Questionnaire used in investigation*	*Results*
**Clark, 2011**	Med Sci Sports Exerc. 2011 Oct;43(10): 1907-12.	n= 121 full-time workers, 18-65 years, ambulatory	GT1M ActiGraph	7 days, over right iliac crest, during waking hours, 1-min epochs, min 4 days	IPAQ, after 7d accelerometer measurement	**Correlation workplace sitting (self-reported) vs. sedentary time at workplace (Acc)**<br>total group<br>r= 0.39, 95% CI = 0.22-0.53 (Pearson correlation)<br>r= 0.29, 95% CI = 0.11-0.44 (Spearman rank-order correlation)<br>**Correlation workplace breaks in sitting time (self-reported) vs. breaks per sedentary hour (Acc)**<br>total group<br>r= 0.26, 95%CI = 0.11-0.44 (Spearman rank-order correlation)
**Lee, 2011**	Int J Behav Nutr Phys Act. 2011 Aug 1;8:81.	n= 1270 (42.9 ± 14.4 years)	ActiGraph GT1M	4 consecutive days, around waist, 2 weekdays+2 weekend days, during waking time, first day always Thursday, Friday, Saturday), less than 600 mins of registered time/day-> invalid, 1 min epoch, moderate(3-5,99MET/1952-5724counts), vigorous (≥6METs/≥5725counts)	IPAQ-C, short form (9items vs. 31items long form), equivalent psychometric properties to the long form	Spearman correlation<br>**Correlation IPAQ-C (moderate PA) vs. Acc (min in moderate PA)**<br>r= 0.05 ≤29 years; r= 0.09 30-49 years; r=0.12, $P<0.05$ ≥50 years<br>**Correlation IPAQ-C (vigorous PA) vs. Acc (min in vigorous PA)**<br>r= 0.21, $P<0.001$ ≤29 years; r= 0.12, $P<0.01$ 30-49 years; r=0.14, $P<0.01$ ≥50 years<br>**Correlation IPAQ-C (total MET) vs. Acc (counts/h)**<br>r= 0.04 ≤29 years; r= 0.19, $P<0.001$ 30-49 years; r=0.25, $P<0.001$ ≥50 years
**Nang, 2011**	BMC Med Res Methodol. 2011 Oct 13;11:141	152 adults (21+ years)	Actical physical activity monitor (Mini Mitter Co., Inc., Bend, OR)	5 consecutive days, right hip with elastic belts, 3 weekdays, 2 weekend days, 15sec epochs, converted to 1min epochs for analysis of energy expenditure	IPAQ, 120 participants before measurement and immediately afterwards, SP2PAQ (Singapore Prospective Study Program PAQu), 43 the other way around	**Correlation IPAQ-Acc:**<br>age ≤ 40:<br>0.08/ 0.09 corrected (moderate),<br>0.30/ 0.52 corrected (vigorous), $p<0.05$<br>age >40:<br>r= 0.21/ 0.24 corrected (moderate),<br>r= -0.07/ -0.01 corrected (vigorous)<br>**Correlation SP2PAQ-Acc**:<br>age ≤ 40:<br>r= 0.21/ 0.24 corrected, $p< 0.05$ (moderate),<br>r= 0.48, $p< 0.0001$/ 0.83 corrected (vigorous), $p<0.05$

Additional File 3. Summary of correlations of physical activity data assessed by questionnaires and accelerometers with respect to Age *(continued)*

Study	*Journal*	*No. of Participants*	*Accelerometer used*	*Accelerometer wear method*	*Questionnaire used in investigation*	*Results*
**Nang, 2011** *continued*	BMC Med Res Methodol. 2011 Oct 13;11:141	152 adults (21+ years)	Actical physical activity monitor (Mini Mitter Co., Inc., Bend, OR)	5 consecutive days, right hip with elastic belts, 3 weekdays, 2 weekend days, 15sec epochs, converted to 1min epochs for analysis of energy expenditure	IPAQ, 120 participants before measurement and immediately afterwards, SP2PAQ (Singapore Prospective Study Program PAQu), 43 the other way around	age >40:<br>r= 0.27/ 0.30 corrected (moderate);<br>r= 0.48, p< 0.0015/ 0.85 corrected (vigorous)<br>*corrected: for within-person variation in acc measurement
**Tomioka, 2011**	J Epidemiol. 2011 Nov 5;21(6):459-65. Epub 2011 Sep 24	325 (164 men, 161 women), >=65 years,	Kenz Lifecorder PLUS, Suzuken Co., Ltd, Nagoya, Japan	Left or right hip, on waist belt, ≤ 2 weeks (preferably 4 weeks), starting on day after 1st IPAQ, 2nd IPAQ on 14th day of accelerometer wear, during waking hours	IPAQ short form, twice, 2 weeks apart, 2 different sets of MET values: 1. original IPAQ values, 2. modified for use with elderly	Spearman correlation<br>**Acc (total PA in METmin/week) vs. IPAQ (total PA in METmin/week)**<br>65-74: r= 0.42 men, 0.49 women<br>75-89: r= 0.53 men, 0.49 women *all P< 0.01*<br>**Acc (total PA in METmin/week) vs. IPAQ (vigorous PA min/week)**<br>65-74: r= 0.25 men, P< 0.05, 0.12 women<br>75-89: r= 0.17 men, 0.17 women<br>**Acc (total PA in METmin/week) vs. IPAQ (moderate PA min/week)**<br>65-74: r= 0.26 men, P< 0.05, 0.13 women<br>75-89: r= 0.05 men, 0.03 women
**Hekler, 2012**	J Phys Act Health. 2012 Feb;9(2):225-36.	N=850, ≥66 years,	ActiGraph, model 7164 and 71256 (Manufacturing Technology Incorporated, Fort Walton, FL)	7 days, during waking hours	Modified Community Health Activities Model Program for Seniors physical activity questionnaire (CHAMPS), past 4 weeks, at least 5 days, at least 8 hours/day	Spearman rank order correlations<br>CHAMPS vs. accelerometer<br>Low-light physical activity ρ= 0.06<br>Hight-light physical activity ρ= 0.27, P<0.0001<br>MVPA ρ= 0.37,P<0.0001<br>Total activity ρ=0.38,P<0.0001

Additional File 3. Summary of correlations of physical activity data assessed by questionnaires and accelerometers with respect to Age *(continued)*

Study	*Journal*	*No. of Participants*	*Accelerometer used*	*Accelerometer wear method*	*Questionnaire used in investigation*	*Results*
**Sabia, 2014**	Am J Epidemiol. 2014 Mar 15;179(6):781-90.	n= 3,975, 60-83 years	GeneActiv; Activinsights Ldt., Cambs, UK)	Wrist-worn, on non-dominant wrist, 9 consecutive days, 24 hours, at least 2 weekdays and 2 weekend days for at least 16 hours per day	Modified version of the Minnesota Leisure Time Physical Activity Questionnaire, 20 items on amount of time spent in: walking, sports, gardening, housework, do-it-yourself activity (building, repairing,..), 2 open-ended questions on "other activities"	Spearman correlation questionnaire-accelerometer (total PA)<br><br>Total: ρ=0.33 (95% CI: 0.30-0.36)<br>Women: ρ= 0.32 (95% CI: 0.26-0.37)<br>Men: ρ= 0.33 (95% CI: 0.30-0.37)<br>60-65 years: ρ=0.30 (95% CI: 0.25-0.35)<br>66-70 years: ρ=0.36 (95% CI: 0.31-0.41)<br>71-75 years: ρ=0.34 (95% CI: 0.27-0.40)<br>76-83 years: ρ=0.26 (95% CI: 0.20-0.33)

Abbreviations: IPAQ, International Physical Activity Questionnaire; Acc, accelerometry; PYTPYQ, past year total physical activity questionnaire; PA, physical activity; BWHS, Black Women's Health Study; Qu, Questionnaire; PAQ-EJ, physical activity questionnaire for elderly Japanese; MET, metabolic equivalent of task; PASE, physical activity scale for the elderly; EPIC, European Prospective Investigation into Cancer and Nutrition; EE, energy expenditure; GPAQ, Global Physical Activity Questionnaire; MVPA, moderate-vigorous physical activity; SP2PAQ, Singapore Prospective Study Program Physical Activity Questionnaire
